# Variance components for bovine tuberculosis infection and multi-breed genome-wide association analysis using imputed whole genome sequence data

**DOI:** 10.1371/journal.pone.0212067

**Published:** 2019-02-14

**Authors:** S. C. Ring, D. C. Purfield, M. Good, P. Breslin, E. Ryan, A. Blom, R. D. Evans, M. L. Doherty, D. G. Bradley, D. P. Berry

**Affiliations:** 1 Teagasc, Animal and Grassland Research and Innovation Centre, Moorepark, Fermoy, Co. Cork, Ireland; 2 School of Veterinary Medicine, University College Dublin, Belfield, Dublin 4, Ireland; 3 Department of Agriculture, Food and the Marine, Dublin 2, Ireland; 4 Irish Cattle Breeding Federation, Highfield House, Bandon, Co. Cork, Ireland; 5 Smurfit Institute of Genetics, University of Dublin, Trinity College, Dublin, Ireland; Western Sydney University, AUSTRALIA

## Abstract

Bovine tuberculosis (bTB) is an infectious disease of cattle generally caused by *Mycobacterium bovis*, a bacterium that can elicit disease humans. Since the 1950s, the objective of the national bTB eradication program in Republic of Ireland was the biological extinction of bTB; that purpose has yet to be achieved. Objectives of the present study were to develop the statistical methodology and variance components to undertake routine genetic evaluations for resistance to bTB; also of interest was the detection of regions of the bovine genome putatively associated with bTB infection in dairy and beef breeds. The novelty of the present study, in terms of research on bTB infection, was the use of beef breeds in the genome-wide association and the utilization of imputed whole genome sequence data. Phenotypic bTB data on 781,270 animals together with imputed whole genome sequence data on 7,346 of these animals’ sires were available. Linear mixed models were used to quantify variance components for bTB and EBVs were validated. Within-breed and multi-breed genome-wide associations were undertaken using a single-SNP regression approach. The estimated genetic standard deviation (0.09), heritability (0.12), and repeatability (0.30) substantiate that genetic selection help to eradicate bTB. The multi-breed genome-wide association analysis identified 38 SNPs and 64 QTL regions associated with bTB infection; two QTL regions (both on BTA23) identified in the multi-breed analysis overlapped with the within-breed analyses of Charolais, Limousin, and Holstein-Friesian. Results from the association analysis, coupled with previous studies, suggest bTB is controlled by an infinitely large number of loci, each having a small effect. The methodology and results from the present study will be used to develop national genetic evaluations for bTB in the Republic of Ireland. In addition, results can also be used to help uncover the biological architecture underlying resistance to bTB infection in cattle.

## Introduction

Bovine tuberculosis (bTB) is an infectious disease of ruminants and wildlife populations that also threatens human health. Clinical signs of bTB in cattle are rarely observed in developed countries due to the rigorous surveillance and culling protocols associated with bTB control and eradication programs. Nevertheless, the costs associated with bTB are excessive even in developed countries; for example, the annual operational costs of the bTB eradication program in the Republic of Ireland was €84 million in 2017 [1]. Following the commencement of the bTB eradication program in the Republic of Ireland, the initial success of the program was rapid; nonetheless, progress in more recent years has dwindled [1–2] and the eradication of bTB has yet to be achieved. Therefore, it is timely that an alternative strategy be explored to complement existing bTB eradication programs and hasten the eradication of bTB internationally. Genetic selection is one such complementary strategy.

Genetic selection for improved health has proved fruitful, particularly in Scandinavian countries where access to phenotypes are plentiful [3]. Although health data are generally not routinely recorded in a central repository in the Republic of Ireland, bTB test results are. These data provide a rich opportunity to explore the inter-animal genetic variability for bTB among Irish cattle as well as to identify regions of the genome associated with bTB infection. Previous studies on bTB infection in Irish [4–7] and UK [8–9] cattle have documented exploitable genetic variability for bTB infection (defined as a binary trait); the genetic standard deviation documented for bTB infection ranges from 0.02 to 0.12 units. The presence of genetic variability among cattle for bTB infection supports the view that genetic selection could be a complementary strategy to existing bTB eradication programs. Nonetheless, consistency is lacking in genomic studies on bTB infection in the regions of the genome putatively associated with bTB infection. For example, putative quantitative trait loci (QTL) pertaining to bTB infection in Holstein cattle have been reported on BTA1 [10], BTA2 [5], BTA6 [11], BTA13 [5], BTA22 [12], and BTA23 [7,10]. Even when the same autosome was documented in two studies for association with bTB [7, 10], the QTL identified to harbor putative single nucleotide polymorphisms (SNP) by Richardson et al. [7] were 28 Mb downstream of the QTL identified on the same autosome by Raphaka et al. [10]. As concluded by Raphaka et al. [10], genomic studies on bTB in cattle are likely limited to the population in which they were estimated; this may be due to differences in the phenotype definition, the methodology used, the population itself, and even the polygenic nature of the trait.

The objectives of the present study were to estimate the variance components for bTB infection, with the view of implementing national genetic evaluations for bTB. A second objective was to identify regions of the bovine genome associated with bTB infection in dairy and beef cattle using imputed whole genome sequence data. To our knowledge, the present study will be the first to complete a genome-wide association for bTB using whole genome sequence data and the first also to consider beef breeds.

## Materials and methods

### Phenotype data

Since the 1950s, the Irish Department of Agriculture Food and the Marine (DAFM) has enforced compulsory testing of all cattle in the Republic of Ireland for bTB, normally caused by *Mycobacterium bovis*. All live cattle are tested at least once per annum using the Single Intradermal Comparative Tuberculin Test (SICTT); cattle that yield a positive SICTT result, termed bTB reactors, are immediately removed from their residing herd and slaughtered. In addition, all cattle slaughtered in Irish abattoirs are visually assessed for the presence of bTB lesions in relevant lymph nodes. Observed lesions in slaughtered cattle that did not fail the SICTT test are further examined in a DAFM laboratory to confirm bTB infection; cattle confirmed with bTB in a DAFM laboratory are termed slaughterhouse cases. A more detailed explanation of the testing protocol for the bTB eradication program in the Republic of Ireland can be found at https://www.agriculture.gov.ie/animalhealthwelfare/diseasecontrol/bovinetb/diseaseeradicationtb/ (Accessed 23 May, 2018).

Any herds that yield either a positive bTB reactor or a slaughterhouse case are, according to the national eradication program, regarded as having a bTB-breakdown. Such herds are restricted from trading and they are required to cull animals that yield any positive test result. During a bTB-breakdown, herds are also required to complete continual testing (at ≥ 60 day intervals) for bTB infection; once two consecutive whole-herd tests return entirely negative results, the bTB-breakdown is complete. The time-periods of herd-level bTB-breakdowns were made available to the present study for the period 01^st^ January 2000 to 10^th^ April 2017, inclusive. Tuberculosis test results for bTB reactors and slaughterhouse cases were also provided; test results included SICTT results for bTB reactors and slaughterhouse cases as well as lesion results, for bTB reactors only, and positive laboratory results for slaughterhouse cases only. Details of test results for cattle that yielded entirely negative tuberculosis results or animals observed with lesions at slaughter but subsequently deemed not to have bTB at laboratory examination were not available to the present study. Nevertheless, information on cattle birth, inter-herd movement, death, slaughter and export events together with pedigree data were available from the Irish Cattle Breeding Federation database; these data were used to determine the animals present in each herd on the date(s) bTB reactor(s) and slaughterhouse case(s) were identified. Of the 41,104 bTB-breakdowns in the present study, 4,031,452 cattle resided in 27,029 herds during at least one bTB-breakdown. There were 150,349 bTB reactors and an additional 8,598 confirmed bTB slaughterhouse cases.

### Phenotype and exposure definition

The bTB phenotype considered in the present study was defined for each animal present during each herd-level bTB-breakdown when a bTB reactor or a slaughterhouse case was identified. Cattle that yielded a positive SICTT, lesion, or laboratory result(s) were coded as one (bTB = 1); otherwise, all other cattle present in the herd during the bTB-breakdown were coded as zero (bTB = 0).

In the present study, potential bTB exposure was defined within each herd-level bTB-breakdown for each management group separately. Following bTB infection, tuberculin reactions are generally detectable within 42 days [13]. As a result, management groups were defined by animal age, sex, and parity, 42 days prior to the date herd-mate(s) yielded a positive result (i.e., bTB = 1); animals not present in the herd at least 42 days prior to the date a bTB-breakdown began were not considered for exposure to bTB. Calves were defined as males or females aged between 42 to 365 days; steers were defined as males aged between 366 days to 908 days (i.e., maximum age limit for abattoir premium slaughter price) that were not registered as service bulls; bulls were defined as males aged ≥ 366 days that were registered to have served a female, otherwise, males aged > 908 days were defined as bulls; heifers were defined as nulliparous females aged between 366 days and 1300 days or primiparous females calved < 42 days; cows were defined as primiparous females calved ≥ 42 days or multiparous females.

Animals within a herd management group were deemed exposed to bTB if 1) any herd-mate was identified with a lesion at slaughter after being removed as a bTB reactor, 2) any herd-mate was identified as a slaughterhouse case, or 3) two or more herd-mates were removed from the herd as bTB reactors.

### Phenotype data edits

Only an animal’s most recent bTB result in a given bTB-breakdown within a management group was considered; 5,146,363 records from 4,008,635 cattle in 27,029 herds remained. A total of 273,730 animals in undefined management groups (e.g., nulliparous females ≥ 1300 days) were not considered further. A further 406,915 animals not present in the herd at least 42 days prior to the start date of a bTB-breakdown were also discarded. Primiparous cows that were recorded to have calved < 545 days (i.e., 15 months; 5,152 cows) or multiparous cows that calved > 666 days (i.e., 22 months; 25,210 cows) from the parity median, which were likely to have been managed differently to the majority of the herd, therefore these cows were also removed. In addition, only animals deemed potentially exposed to bTB were retained. Heterosis and recombination loss coefficients were derived using methods described by VanRaden and Sanders [14]. Pedigree information for each animal was traced back to founder animals (where possible) and animals were assigned a genetic group based on breed. Only animals with a known sire were retained. Only completed bTB-breakdowns per management group which comprised of at least five animals and ≥ 1 positive result (i.e., bTB = 1) were considered in the final analyses. After edits, 914,833 records from 781,270 animals remained in 24,893 completed bTB-breakdowns.

### Estimation of variance components

For computational reasons only, the estimation of variance components only considered bTB-breakdowns that were initiated and terminated between the years 2010 and 2017, inclusive. Also for computational reasons, only records from sires that produced at least 10 progeny were considered. Thereafter, a random sample of 3,000 bTB-breakdowns which included 156,467 phenotypes from 144,474 animals were analyzed. Variance components for bTB were estimated using an animal linear mixed model in ASReml version 3.0 [15]. All herd-management groups were considered together in the same analysis. The fitted model was similar to that used by Richardson et al. [6] in a previous analysis of bTB data in Irish cattle:

bTB=μ+HBD+het+rec+sex+lifestage|age+a+PE+e

where *bTB* = the binary dependent variable of the bTB phenotype (i.e., bTB = 0 or bTB = 1); *μ =* the fixed effect of the population mean*; HBD* = the fixed effect of the herd breakdown; *het* = the fixed effect of heterosis coefficient (i.e., 0.00, 0.01 to 0.09, 0.10 to 0.19, … 0.90 to 0.99 and 1.00)*; rec* = the fixed effect of recombination loss coefficient (i.e., 0.00 to 0.09, 0.10 to 0.29, 0.30 to 0.49 and ≥ 0.50); *sex* = the fixed effect of the animal gender; *life stage|age* = the fixed effect of the interaction between animal life stage (i.e., 1 = calves, 2 = heifers and steers, 3 = first parity cows, 4 = second parity cows, 5 = third parity cows, 6 = fourth parity cows, 7 = fifth parity or older cows, 8 = bulls) and animal age fitted as a class effect (i.e., nulliparous animals: age when the final positive test result was generated; cows: days calved when the final positive test result was generated); *a* = the random additive genetic effect, where a ~ *N*(0, **A** σ^2^_a_) with σ^2^_a_ representing the additive genetic variance of the animal and **A** as the additive genetic relationship matrix among animals; *PE* = the random permanent environment effect of the animal, where PE ~ *N*(0, **I** σ^2^_pe_) with σ^2^_pe_ representing the permanent environmental variance and ***I*** an identity matrix; *e* = the random residual effect, where e ~ *N*(0, **I** σ^2^_e_) with σ^2^_e_ representing the residual variance. The coefficient of genetic variation was calculated using the formula by Burdon [16] to adjust for the binary nature of the trait.

### Validation of EBVs

MiX99 (release VIII/2015 version 15.0811) [17] was used to estimate breeding values (**EBV**) for bTB for all 781,270 animals in completed bTB-breakdowns. The model fitted in the genetic evaluation was the same as that described for estimating variance components; the resulting variance components from the present study were used to inform the genetic evaluations.

Phenotypes for bTB were masked (i.e., set to missing) for all animals in a random 20% of herds while pedigree information for those animals were included in the analysis to estimate their breeding values. Both phenotypic and pedigree information for all other animals were included to estimate breeding values. The masked animals were subsequently categorized into one of three risk groups (i.e., low risk, average risk, or high risk) based only on their (parental average) EBV. The odds of each animal yielding a positive bTB diagnosis in their lifetime was calculated per risk group using logistic regression which included a fixed effect representing herd, life stage, and age (as well as EBV risk group); only animals with an EBV reliability ≥ 10% were considered in the logistic regression. This validation process was iterated five times, such that each animal’s phenotype was masked at least once to generate its EBV. The ability of the EBV risk group to determine whether or not each masked animal was diagnosed with bTB infection during their lifetime were also measured by the area under the receiver operating curve where the EBV risk group and life stage were the other terms included in the model; only animals with an EBV reliability ≥10% were again considered. Based on random chance alone, the area under the receiver operating curve would be 0.5, whereas if the model were perfectly able to determine the phenotype of each animal, the area under the receiver operating curve would be 1.0 [18].

### Genotype data

Of the cattle in the edited phenotype dataset, 14,778 of those animals’ sires were genotyped. The majority of the genotyped bulls were purebred Charolais (5,390), Limousin (4,539), Holstein-Friesian (1,696), Aberdeen Angus (651), or Simmental (633) breeds. Each bull was genotyped using either a high-density genotype panel (777,962 SNPs; 3,354 bulls), a Bovine SNP50 panel (54,001 SNPs; 721 bulls), or a custom built International Dairy and Beef (IDB) genotyping panel (version 1 including 17,137 SNPs: 461 bulls; version 2 including 18,004 SNPs: 9,463 bulls; version 3 including 53,450 SNPs 779 bulls). Each bull had a call rate of at least 90%. Only autosomal SNPs, which had a known chromosome and position with a SNP call rate of at least 90% within panel was retained.

#### Imputation

All genotyped bulls were imputed to HD using a two-step approach in FImpute2 [19]; this involved imputing the IDB genotyped bulls to the Bovine SNP50 density and consequently imputing all resulting genotypes (including the Bovine SNP50 genotypes) to HD using a population of 5,504 influential sires from multiple breeds genotyped on the HD. Imputation to WGS was then undertaken using a reference population of 2,333 *Bos taurus* animals of multiple breeds from Run6.0 of the 1000 Bulls Genomes Project [20]. All variants in the reference population were called using SAMtools version 1.3.1 and genotype calls were improved using Beagle software version 4.1 to provide a consensus SNP density across all animals. Details of alignment to UMD 3.1, variant calling and quality controls completed within the multi-breed reference population are described by Daetwyler et al. [20]. In total, 41.39 million SNP variants were identified across the genome and the average coverage was 12.85X.

Imputation of the HD genotypes to WGS was then completed by first phasing all imputed HD genotypes using Eagle version 2.3.2 [21] and subsequently imputing to WGS using minimac3 [22]. To assess the accuracy of imputation to WGS, a validation set of 175 animals that were genotyped on either the Bovine SNP50 or HD genotype panels, as well as being sequenced, were used. This involved using the animal’s original genotype panel calls, and imputing these genotypes to WGS using both Eagle and minimac3, whilst removing the validation animals from the reference WGS population. The average genotype concordance, defined as the proportion of correctly called SNPs across the genome versus all SNPs, was estimated to be 0.98 in the 175 animals. After imputation, a total of 41,389,526 SNPs existed for all bulls.

Regions of poor WGS imputation accuracy, perhaps due to local mis-assemblies or mis-orientated contigs, were identified using an additional dataset of 147,309 verified parent-progeny relationships. Mendelian errors, defined as the proportion of opposing homozygotes in a parent-progeny pair, were estimated for each relationship and the subsequent Mendelian error rate per SNP was determined. To accurately identify genomic regions of poor imputation, the R package GenWin [23] which fits a β-spline to the data to find likely inflection points, was used to identify genomic region breakpoints of high Mendelian errors. Windows were analyzed using an initial window size of 5 Kb and Genwin pooled windows, for which the SNP Mendelian error rate were similar. The average SNP Mendelian error rate per window was estimated and all SNPs within windows where the mean SNP Mendelian error rate was >0.02 were removed.

### Genome-wide association analysis

#### Phenotype

MiX99 (release VIII/2015 version 15.0811) [17] was used to derive EBVs for bTB for all 781,270 animals in completed bTB-breakdowns; both phenotypic and pedigree information for all animals in the edited phenotype dataset were included to generate EBVs for the subsequent genome-wide associations of these animals’ sires but also to determine genetic trends. For the 14,778 genotyped bulls, EBVs were deregressed in MiX99 using the Secant method [24]. Deregressed EBVs were weighted using the formula by Garrick et al. [25]:

$$w_{i}=\frac{1-h^{2}}{\left[c+\frac{1-r_{i}^{2}}{r_{i}^{2}}\right]h^{2}}$$

where *w_i_* = is the weighting factor of the deregressed EBV of the *i*th animal, *h*^2^ is the heritability estimate (i.e., h^2^ = 0.12 as estimated in the present study), $r_{i}^{2}$ is the reliability of the deregressed EBV for the *i*th animal, and *c* is the genetic variance not accounted for by the SNPs (i.e., c = 0.90).

**Fig 1:**
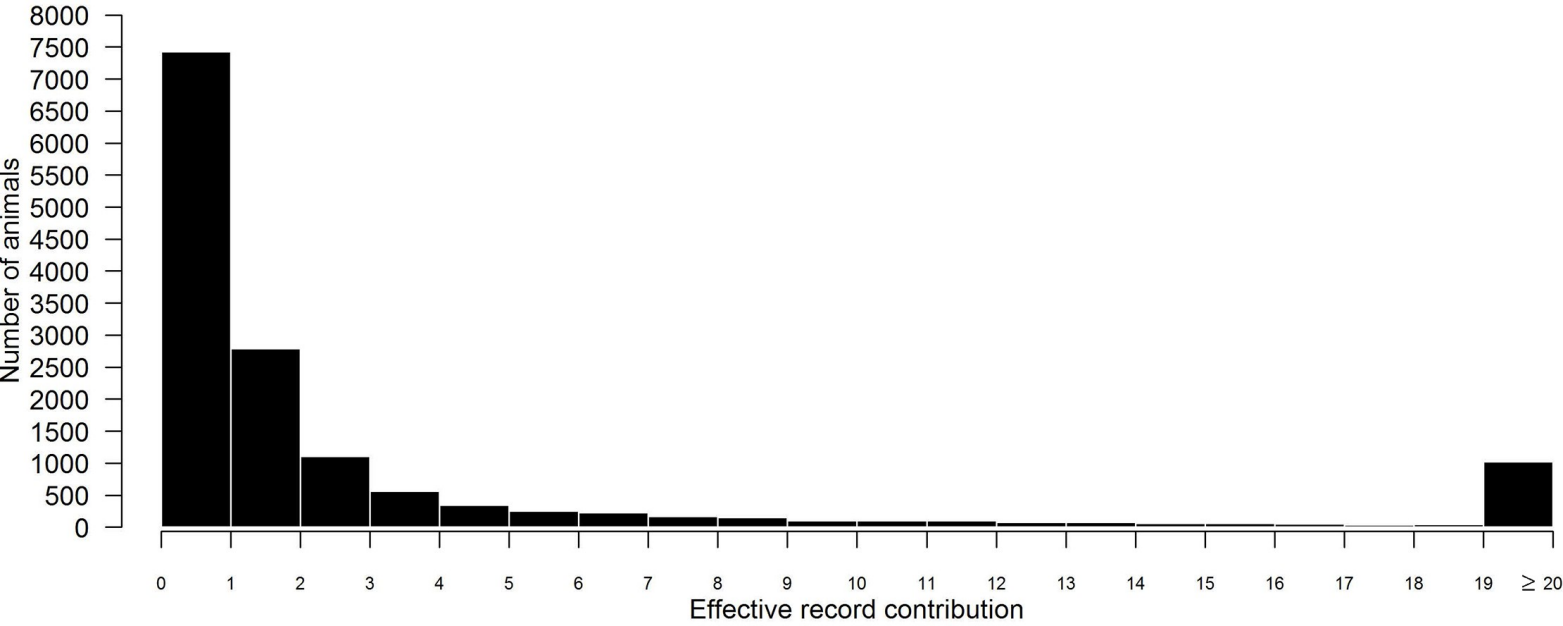
The effective record contribution of the 14,778 genotyped bulls.

Only the 7,346 bulls that had genotype data with an effective record contribution (ERC) of at least one (median ERC for the 7,346 bulls = 2.7) were considered further (Fig 1); of these bulls, there were 2,039 Charolais (median ERC = 1.9), 1,964 Limousin (median ERC = 2.1), 1,502 Holstein-Friesian (median ERC = 13.9), 382 Aberdeen Angus (median ERC = 2.4), and 328 Simmental (median ERC = 3.2) purebred bulls; the remaining 1,131 bulls (median ERC = 2.9) were of another purebred beef or dairy breed, or of a mixed breed. For the 7,346 remaining bulls (each of which were considered in the one multi-breed analysis), SNPs with a minor allele frequency (MAF) < 0.002 or SNPs that deviated from Hardy-Weinberg equilibrium (P < 10^−6^) were removed; 10,506,082 autosomal SNPs remained. For the within-breed analyses of the larger purebred populations of Charolais, Limousin, and Holstein-Friesian bulls, SNPs with a within-breed minor allele frequency (MAF) < 0.002 or SNPs that deviated from Hardy-Weinberg equilibrium (P < 10^−6^; calculated within-breed) were removed; 17,250,600, 17,267,260, and 15,017,692 autosomal SNPs remained for the Charolais, Limousin, and Holstein-Friesian analysis, respectively.

#### Single-SNP regression

Using only autosomal HD genotypes, the genomic relationship matrix was constructed between bulls using Van Raden method 1 [26]. SNP effects were estimated singly using mixed models in WOMBAT (most up-to-date version on 23/02/2018) [27–28] for all 7,346 bulls in the one analysis and separately three within-breed analyses were also completed for the 2,039 purebred Charolais bulls, the 1,964 purebred Limousin bulls, and the 1,502 purebred Holstein-Friesian bulls. Except for the fixed effect of *breed* which was not included in the within-breed analyses, the model fitted in all analyses was:

$$EBV_{j}=\mu +SNP_{j}+breed_{j}+a_{j}+e_{j}$$

*w*here ***EBV***_***j***_ = the dependent variable of the deregressed EBV weighted by ERC of the *j*th individual; ***μ*** *=* the fixed effect of the population mean; ***SNP***_***j***_ = the fixed effect of allele dosage for each SNP (coded as 0, 1, or 2) of the *j*th individual; ***breed***_***j***_ = the fixed effect of breed percentage (i.e., ranging from 0 to 100) fitted as a covariate (Aubrac, Blonde d'Aquitaine, Belgian Blue, Charolais, Hereford, Limousin, Saler, Simmental, Shorthorn, Parthenaise, Ayrshire, Brown Swiss, Friesian, Jersey, Montbéliarde, Holstein, Norwegian Red, Normandé, and “Unknown”; Aberdeen Angus was not fitted in the model to avoid linear dependencies) of the *j*th individual; ***a***_***j***_ is the random effect of the *j*th individual; ***e***_*j*_ = the residual effect of the *j*th individual, e_j_ ∼ *N*(0, σ_e_^2^), with σ_e_^2^ being the residual variance. The genome-wide and suggestive significance threshold were defined as P = 1 x 10^−6^ and P = 1 x 10^−5^, respectively.

#### Defining QTLs

Within each analysis, QTL regions associated with bTB infection were defined based on linkage disequilibrium (LD) patterns with SNPs that surpassed the suggestive significance threshold (P < 1 x 10^−5^). Pairwise LD was calculated as the r^2^ among all SNPs within 5 Mb of the suggestive SNPs; the beginning and end of each QTL were defined by the SNPs that were furthest upstream and downstream of the suggestive SNP that had an r^2^ of ≥ 0.5. Overlapping QTL were merged together and considered as the same QTL.

#### Identifying candidate genes

The defined QTL regions were subsequently explored for the presence of candidate genes using ENSEMBL (https://www.ensembl.org/) on the UMD 3.1 genome build. A pathway analysis was undertaken using InnateDB [29] using the hypergeometric algorithm and the Benjamin Hochberg correction; pathways specific to cattle were initially considered, then pathways predicted by orthology to humans for association with bTB infection were also considered.

## Results

### Descriptive statistics, fixed effects and variance components

**Fig 2:**
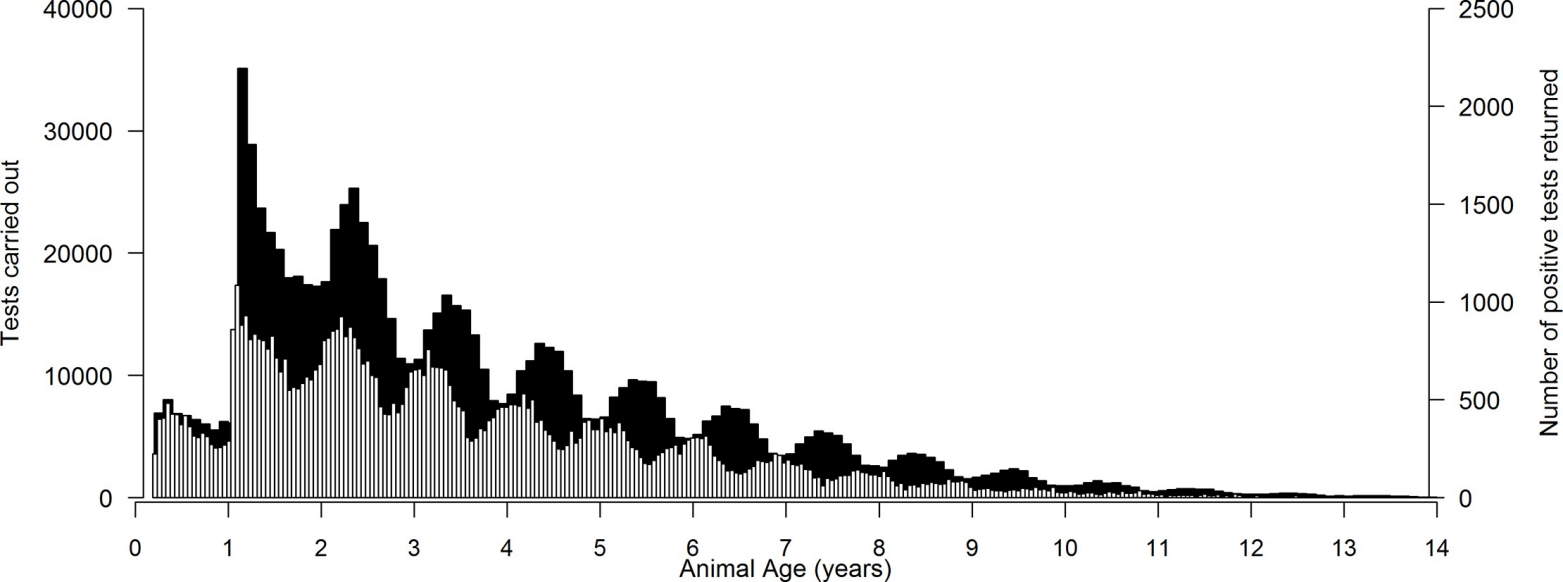
Distribution by animal age (in years) of the total number of tests carried out (primary axis; filled) and the number of positive results returned (secondary axis; no fill) during bovine tuberculosis-breakdowns.

Cows (61%), followed by heifers (19%), represented the majority of the dataset after edits, with <1.5% of the dataset being bulls. After edits, the mean prevalence of bTB in the entire dataset was 7.8%. More positive tests originated from younger animals (i.e., 1 to 2 year olds) compared to older animals (Fig 2), but the total number of tests undertaken on younger animals was higher than that from older animals. The fixed effects of life stage and age, together with their interaction, were associated (P < 0.001) with bTB diagnosis, as was animal gender (P < 0.01). Of the different life stages, bulls and cows had the highest odds of being diagnosed with bTB infection, whereas calves had the least odds of being diagnosed with bTB infection (Table 1). Additionally, as nulliparous age increased, or as the stage of lactation in calved cows progressed, the odds of a positive bTB diagnosis also increased (P < 0.001).

**Table 1:** Associations between the life stage of the animal on the odds ratio of a positive bTB diagnosis (95% CI in parentheses) relative to the referent life stage of a calf.

Life stage	Odds ratio
Heifers and steers	1.06 (1.04 to 1.07)
1^st^ parity cows	1.11 (1.09 to 1.13)
2^nd^ parity cows	1.14 (1.12 to 1.16)
≥3^rd^ parity cows	1.15 (1.13 to 1.17)
Bulls	1.33 (1.27 to 1.39)

**Fig 3:**
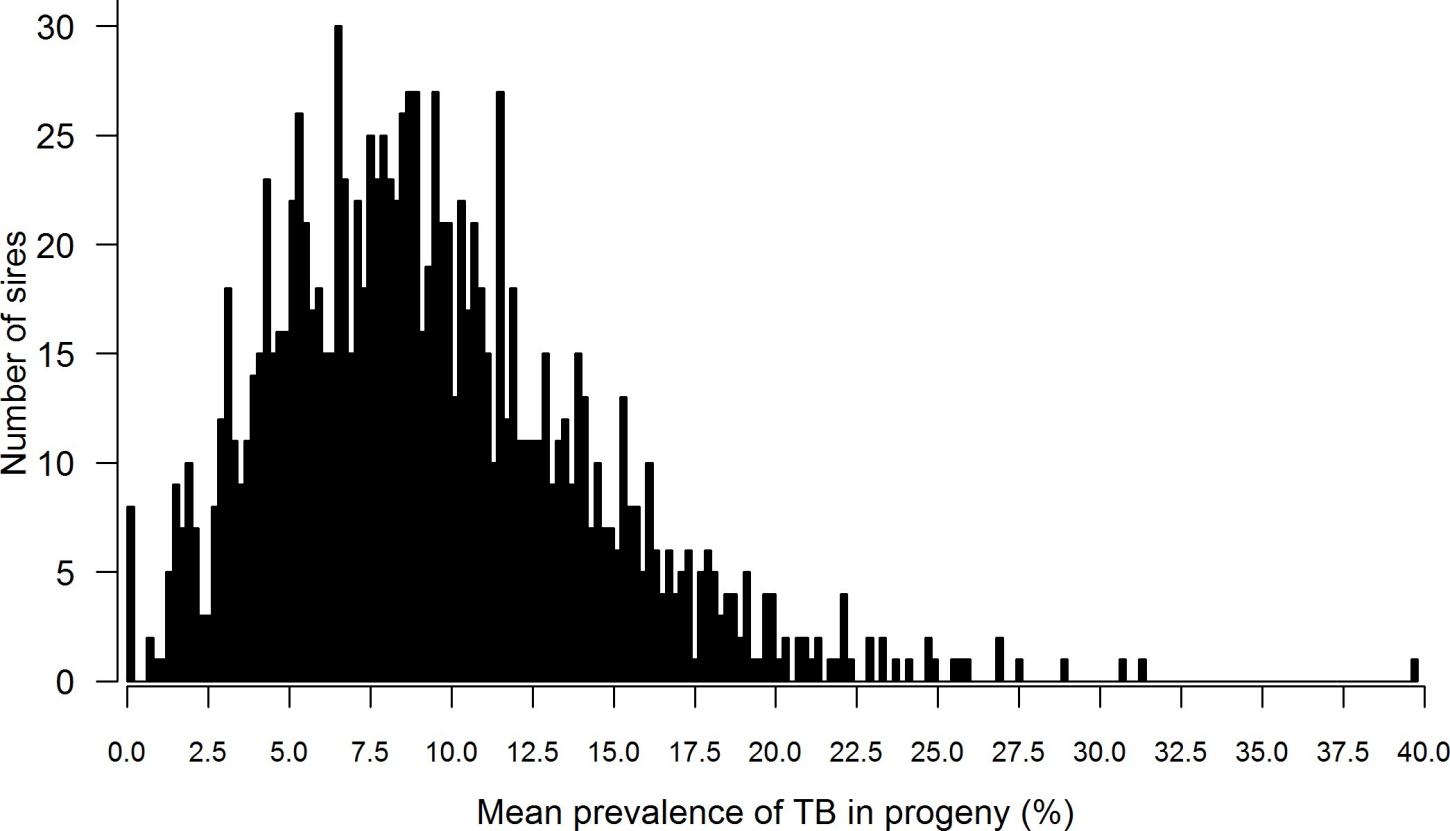
Distribution of the mean prevalence of bovine tuberculosis in the progeny of 1,262 sires that produced at least 50 progeny in 10 bovine tuberculosis-breakdowns.

Illustrated in Fig 3 is a distribution of the average prevalence of bTB in the progeny of sires, which had ≥ 50 progeny in ≥ 10 herds deemed exposed to bTB. For most sires, the average prevalence of bTB in the progeny of sires was 10% or less; nonetheless, for three sires, of which two were Charolais (67 progeny in 49 herds and 75 progeny in 62 herds) and one was Holstein-Friesian (73 progeny in 30 herds), the average prevalence of bTB in their progeny ranged from 31% to 40% which is indicative of underlying genetic variability to bTB. For bTB, the genetic standard deviation was 0.09, the heritability estimate was 0.12 (SE = 0.007), the repeatability estimate was 0.30 (SE = 0.010), and the coefficient of genetic variation was 32%.

### Genetic trends

**Fig 4:**
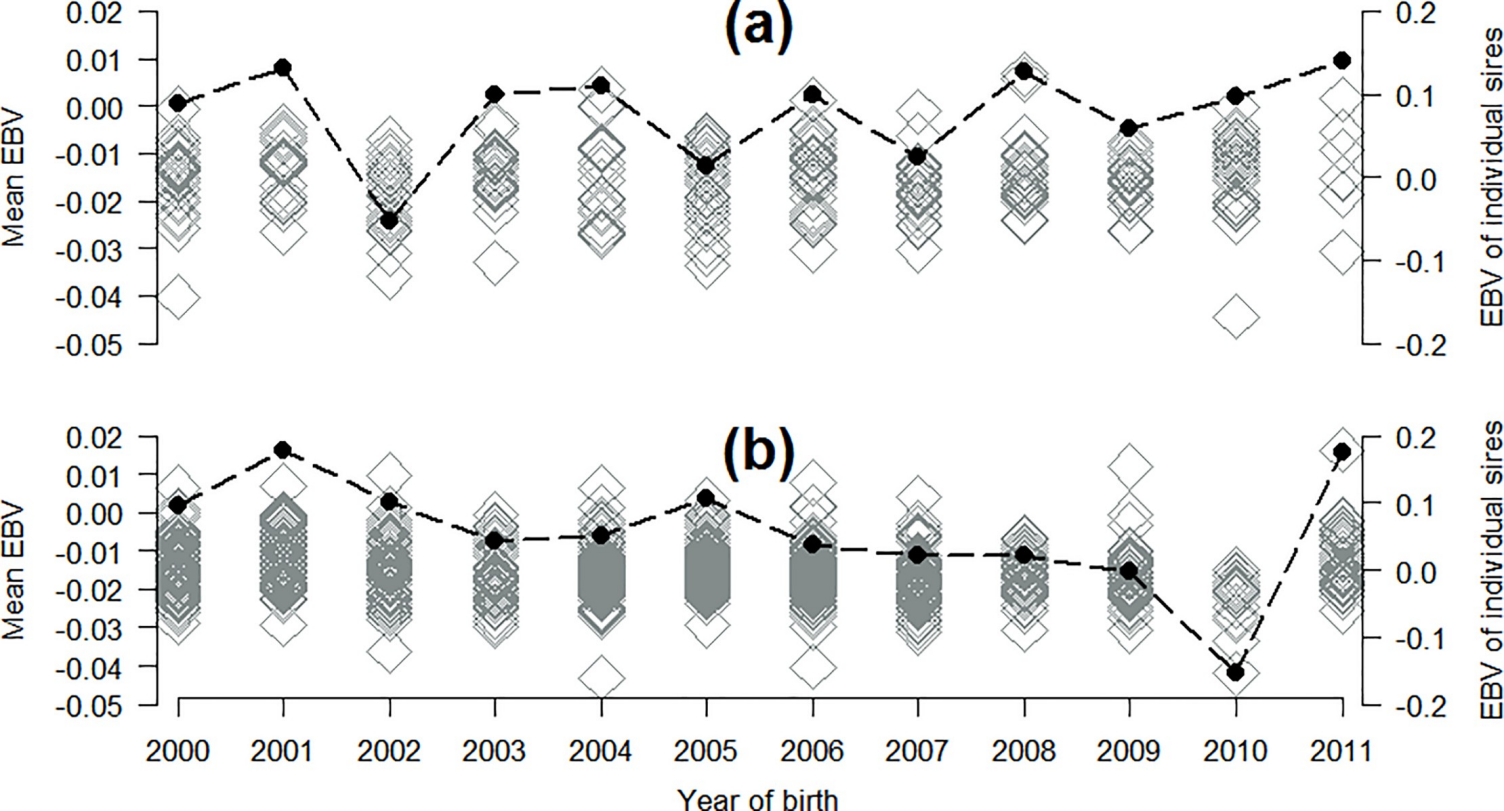
Distribution of the mean sire EBV (primary axis; line) and individual sire EBV (secondary axis; diamond) per year of birth for a) 243 beef AI sires and b) 799 dairy AI sires that had ≥ 20 progeny with a bovine tuberculosis phenotype in at least 2 herds.

Genetic trends for AI sires born between the years 2000 and 2011 that had at least 20 progeny in at least two bTB-breakdowns are in Fig 4. While a large fluctuation in the mean EBV may be perceived, it should be noted that the range of mean EBVs in Fig 4 is relatively narrow (-0.05 to +0.02). Moreover, the mean statistic is heavily influence by outliers which explains the reason for the perceived large fluctuation in mean EBV. Although not different from zero for beef breeds, the mean EBV was higher (i.e., worse; on average 0.007 EBV units higher) for beef breeds than for dairy breeds across all years (Fig 4). The slope of the linear regression coefficient of mean EBV per year of birth for the years 2000 and 2011 for beef AI sires was -0.0006 (SE = 0.00059) and the slope was -0.0020 (SE = 0.00040) for dairy AI sires.

### Validation of EBVs

When only the parental average EBV of the animal was considered for predicting bTB infection in bTB-breakdown herds, the mean bTB EBV reliability for animals was 19% and the area under the receiver operating curve was 0.529 (95% CI: 0.5236 to 0.5342); when the life stage of the animal was also known, the area under the receiver operating curve increased to 0.555 (95% CI: 0.5494 to 0.5612). The odds of yielding a positive bTB result increased (P < 0.001) with bTB EBV risk group, when herd and life stage were accounted for. Cattle in the worst 20^th^ percentile for bTB EBV had a 1.44 (95% CI: 1.34 to 1.54) times greater odds of returning a positive bTB result compared to cattle in the best 20^th^ percentile for bTB EBV; cattle with an intermediate bTB EBV (i.e., 21^st^ to 79^th^ percentile) had, on average, a 1.20 (95% CI: 1.13 to 1.28) times greater odds of returning a positive bTB result compared to cattle in the best 20^th^ percentile for bTB EBV. Phenotypically, the high risk EBV group (mean prevalence of 9.3%) had a 1.8 percentage unit higher prevalence of positive bTB results compared to the average risk EBV group (mean prevalence of 7.5%); whereas the high risk EBV group had a 2.45 percentage unit higher prevalence of positive bTB results compared to the low risk EBV group (mean prevalence of 6.9%).

### Multi-breed genome-wide association

**Fig 5:**
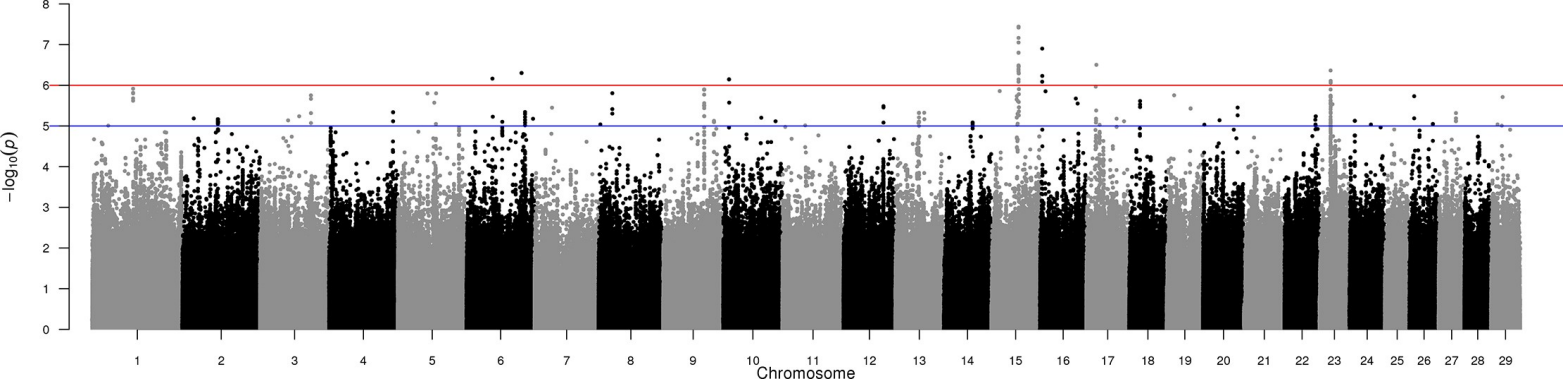
Manhattan plot showing–log_10_ (P-values) of association between each single nucleotide polymorphism effect and the weighted deregressed EBV for bovine tuberculosis infection in the multi-breed analysis of 7,346 bulls; red and blue lines represent genome-wide and suggestive thresholds, respectively.

A Manhattan plot outlining the association between each SNP and the EBV for bTB infection in the multi-breed analysis is in Fig 5. A corresponding QQ-plot of the observed P-values compared to the expected P-values is in S1 Fig. Thirty-eight SNPs were significantly associated with bTB infection at the genome-wide threshold of P = 1 x 10^−6^ (Table 2). Of these, 19 SNPs were located in the one QTL region on BTA15, spanning from 47.66 Mb to 47.77 Mb; this region included just five genes, each of which were associated with olfactory receptors (*ENSBTAG00000048223*, *ENSBTAG00000047246*, *ENSBTAG00000032280*, *ENSBTAG00000023125*, and *OR52E5*). The three SNPs with the strongest (P = 3.63 x 10^−8^) association, rs42709904, rs799252319, and rs42898143 were all located on BTA15 where the minor allele A (multi-breed frequency: 0.005) was associated with increased resistance to bTB infection; two of these SNPs were classified as synonymous or upstream gene variants of the gene *OR52E5*. Notably, 6 of the 9 SNPS categorized as missense gene variants of *OR52E5* were segregating yet the P-values were ≥ 0.32. For each of the 38 SNPs with a P-value < 1 x 10^−6^ (Table 2), where the frequency of the favorable allele in the entire dataset was low (< 0.005), the frequency of the favorable allele in each of the seven main breeds represented in the dataset was also low or absent (frequency range of the seven predominant breeds: 0.000 to 0.105), and *vice versa* (S1 Table). Located within the gene *RCAN2*, the major C allele (multi-breed frequency: 0.765) of SNP rs133701091, which had the lowest P-value of all SNPs on BTA23 (P = 4.3 x 10^−7^), was associated with increased resistance to bTB infection.

**Table 2:** Chromosome (BTA), position, P-value, the favorable allele, the frequency of the favorable allele, substitution effect of the favorable allele, annotation, and gene for the 38 single nucleotide polymorphisms associated with bovine tuberculosis infection (P < 1 x 10^−6^).

BTA	Position	P-value	Allele	Frequency	Effect	Annotation	Gene
15	47754771	3.63x10^-8^	A	0.005	0.060	synonymous	*OR52E5*
15	47755338	3.63x10^-8^	A	0.005	0.060	upstream gene	*OR52E5*
15	47765272	3.63x10^-8^	A	0.005	0.060	intergenic	* *
15	47752145	3.82x10^-8^	C	0.005	0.061	downstream gene	*OR52E5*
15	47695516	6.84x10^-8^	T	0.005	0.058	intergenic	* *
15	47729320	8.87x10^-8^	T	0.004	0.064	intergenic	
16	4080552	1.25x10^-7^	A	0.984	0.037	intron	*SRGAP2*
15	47735420	1.57x10^-7^	A	0.004	0.059	synonymous	*ENSBTAG00000023125*
15	47688637	1.59x10^-7^	C	0.005	0.056	upstream gene	*ENSBTAG00000047246*
17	17595339	3.14x10^-7^	A	0.997	0.077	intron	*CLGN*
15	47738084	3.25x10^-7^	A	0.004	0.060	upstream gene	*ENSBTAG00000023125*
15	47740797	3.25x10^-7^	T	0.004	0.060	intergenic	* *
15	47683747	3.41x10^-7^	A	0.005	0.055	synonymous	*ENSBTAG00000047246*
15	47750477	3.44x10^-7^	A	0.004	0.060	downstream gene	*OR52E5*
15	48159563	3.44x10^-7^	T	0.004	0.061	intergenic	* *
15	47655527	3.98x10^-7^	C	0.005	0.055	intergenic	* *
15	47657277	3.98x10^-7^	T	0.005	0.055	intergenic	* *
15	47682180	4.14x10^-7^	G	0.005	0.055	downstream gene	*ENSBTAG00000047246*
23	19465559	4.32x10^-7^	C	0.765	0.010	intron	*RCAN2*
15	47671329	4.56x10^-7^	A	0.005	0.055	upstream gene	*ENSBTAG00000048223*
15	47672311	4.56x10^-7^	T	0.005	0.055	upstream gene	*ENSBTAG00000048223*
6	96523708	4.98x10^-7^	G	0.996	0.049	intron	*ANTXR2*
6	96544980	4.98x10^-7^	G	0.996	0.049	intergenic	* *
15	48067616	5.09x10^-7^	T	0.004	0.063	intergenic	* *
15	48070477	5.09x10^-7^	G	0.004	0.063	downstream gene	*ENSBTAG00000023912*
15	48075512	5.09x10^-7^	G	0.004	0.063	upstream gene	*ENSBTAG00000023912*
16	4168671	5.89x10^-7^	G	0.985	0.036	intron	*RASSF5*
6	45305344	6.85x10^-7^	T	0.994	0.049	intergenic	* *
10	10238600	7.15x10^-7^	G	0.994	0.052	upstream gene	*JMY*
10	10268287	7.15x10^-7^	C	0.994	0.052	intron	*JMY*
15	47751834	7.19x10^-7^	C	0.004	0.058	downstream gene	*OR52E5*
23	19657604	7.79x10^-7^	C	0.887	0.013	intron	*RCAN2*
23	19632067	7.98x10^-7^	G	0.888	0.013	intron	*RCAN2*
23	19634225	7.98x10^-7^	C	0.888	0.013	intron	*RCAN2*
16	4041643	8.17x10^-7^	G	0.985	0.036	intron	*SRGAP2*
15	47682642	8.17x10^-7^	A	0.005	0.053	downstream gene	*ENSBTAG00000047246*
23	19642280	8.24x10^-7^	G	0.888	0.013	intron	*RCAN2*
23	19641604	8.70x10^-7^	G	0.872	0.012	intron	*RCAN2*

Fifty-three QTL regions included ≥1 SNP that flanked 5 Mb of a suggestive SNP (P < 1 x 10^−5^) with an r^2^ of ≥ 0.5. Those QTL ranged from 8 bp to 5. 9 Mb in length and were identified across 26 autosomes based on the multi-breed analysis. For a further 11 suggestive SNPs (P < 1 x 10^−5^), the QTL included only the suggestive SNP itself as no other SNP existed within 5 Mb with an r^2^ of ≥ 0.5. A list of all QTLs is in S2 Table. QTLs that contained at least six suggestive SNPs (P < 1 x 10^−5^) associated with bTB infection are in Table 3. From the 273 genes identified in the 64 QTL regions, thirteen biological pathways specific to cattle were identified (Table 4) while 157 pathways predicted by orthology to humans were also deemed to be associated with bTB infection (Table 5).

**Table 3:** Chromosome (BTA), start position, end position, number of suggestive single nucleotide polymorphisms (SNPs), and the genes identified in quantitative trait loci that had at least six suggestive single nucleotide polymorphisms (P < 1 x 10^−5^) associated with bovine tuberculosis infection.

BTA	Start	End	SNPs	Genes in quantitative trait loci
2	62187019	62778428	50	*ZRANB3*, *RAB3GAP1*, *MAP3K19*, *ENSBTAG00000048182*, *CCNT2*, *ACMSD*
6	102640308	102691067	6	*ARHGAP24*, *MAPK10*
9	72193061	72247962	19	
13	35848759	41746215	12	*BAMBI*, *WAC*, *MPP7*, *ARMC4*, *MKX*, *RAB18*, *ENSBTAG00000048079*, *PCSK2*, *ENSBTAG00000006043*, *BFSP1*, *DSTN*, *ENSBTAG00000015438*, *BANF2*, *SNX5*, *RF00567*, *MGME1*, *OVOL2*, *KAT14*, *ZNF133*, *DZANK1*, *POLR3F*, *RBBP9*, *SEC23B*, *DTD1*, *ENSBTAG00000012276*, *RF00001*, *SCP2D1*, *SLC24A3*, *ENSBTAG00000032893*, *RIN2*, *bta-mir-2305*, *NAA20*, *CRNKL1*, *CFAP61*, *INSM1*, *RALGAPA2*, *RF00156*, *KIZ*,*XRN2*, *NKX2-4*, *NKX2-2*, *ENSBTAG00000035643*, *PAX1*
15	44461212	46371067	8	*RPL27A*, *RF00334*, *RF00334*, *TRIM66*, *STK33*, *LMO1*, *RIC3*, *TUB*, *EIF3F*, *ENSBTAG00000039405*, *ENSBTAG00000048045*, *ENSBTAG00000040252*, *ENSBTAG00000038641*, *OR10A6*, *ENSBTAG00000021150*, *OR5P3*, *ENSBTAG00000040285*, *ENSBTAG00000048176*, *ENSBTAG00000045639*, *ENSBTAG00000037384*, *ENSBTAG00000026826*, *ENSBTAG00000038317*, *ENSBTAG00000038245*, *ENSBTAG00000047850*, *ENSBTAG00000038598*, *OVCH2*, *RF00026*, *CYB5R2*, *PPFIBP2*, *OLFML1*, *SYT9*, *ENSBTAG00000046099*, *RBMXL2*, *NLRP14*, *ZNF214*
15	47655527	47765272	19	*ENSBTAG00000048223*, *ENSBTAG00000047246*, *ENSBTAG00000032280*, *ENSBTAG00000023125*, *OR52E5*
15	48067616	48400758	11	*ENSBTAG00000023912*, *ENSBTAG00000026929*, *ENSBTAG00000027889*, *ENSBTAG00000026924*, *ENSBTAG00000039029*, *ENSBTAG00000046618*, *ENSBTAG00000026922*, *ENSBTAG00000016119*, *ENSBTAG00000006815*, *ENSBTAG00000046764*, *ENSBTAG00000047088*, *ENSBTAG00000005609*, *ENSBTAG00000000953*
23	19441898	19592407	24	*RCAN2*, *ENSBTAG00000022736*
23	19617330	19662590	16	*RCAN2*
27	30187171	30278015	7	

**Table 4:** Bovine biological pathways associated with bovine tuberculosis infection.

Pathway name	Corrected p-value	Genes identified in pathway
ATF6-alpha activates chaperone genes	0.0038	*HSP90B1*, *NFYB*
Metabolism	0.0040	*ACMSD*, *BHMT*, *CHPT1*, *MGST2*, *NDUFC1*, *NFYB*, *PAH*, *PPAP2B*, *SACM1L*, *SCARB1*, *TXNRD1*
ATF6-alpha activates chaperones	0.0040	*HSP90B1*, *NFYB*
Metabolism of lipids and lipoproteins	0.0048	*CHPT1*, *NFYB*, *PPAP2B*, *SACM1L*, *SCARB1*, *TXNRD1*
PPARA activates gene expression	0.0378	*NFYB*, *TXNRD1*
Regulation of lipid metabolism by Peroxisome proliferator-activated receptor alpha	0.0378	*NFYB*, *TXNRD1*
Binding and Uptake of Ligands by Scavenger Receptors	0.0390	*HSP90B1*, *SCARB1*
Metabolism of amino acids and derivatives	0.0436	*ACMSD*, *BHMT*, *PAH*
Unfolded Protein Response (UPR)	0.0454	*HSP90B1*, *NFYB*
Phospholipid metabolism	0.0498	*CHPT1*, *SACM1L*
Fatty acid, triacylglycerol, and ketone body metabolism	0.0774	*NFYB*, *TXNRD1*
Metabolism of proteins	0.3451	*HSP90B1*, *IGF1*, *NFYB*
Signal Transduction	0.9046	*ARHGAP29*, *IGF1*

**Table 5:** Top ten biological pathways associated with bovine tuberculosis infection predicted by orthology from humans.

Pathway name	Corrected p-value	Genes identified in pathway
Protein processing in endoplasmic reticulum	0.0547	*CUL1*, *ERP70*, *HSP90B1*, *MAPK10*, *RRBP1*, *SEC23B*
ATF6-alpha activates chaperone genes	0.0579	*HSP90B1*, *NFYB*
Organic anion transporters	0.0579	*SLC17A8*, *SMCT1*
ATF6-alpha activates chaperones	0.0614	*HSP90B1*, *NFYB*
Alternative complement pathway	0.0614	*BT*.*19507*, *BT*.*39896*
Terminal pathway of complement	0.0656	*BT*.*19507*, *BT*.*39896*
Transport of inorganic cations/anions and amino acids/oligopeptides	0.0723	*SLC17A8*, *SLC24A3*, *SLC6A20*, *SLC7A11*, *SMCT1*
Classical complement pathway	0.0728	*BT*.*19507*, *BT*.*39896*
Lectin induced complement pathway	0.0728	*BT*.*19507*, *BT*.*39896*
Regulation of Complement cascade	0.0909	*BT*.*19507*, *BT*.*39896*

### Within-breed genome-wide association

**Fig 6:**
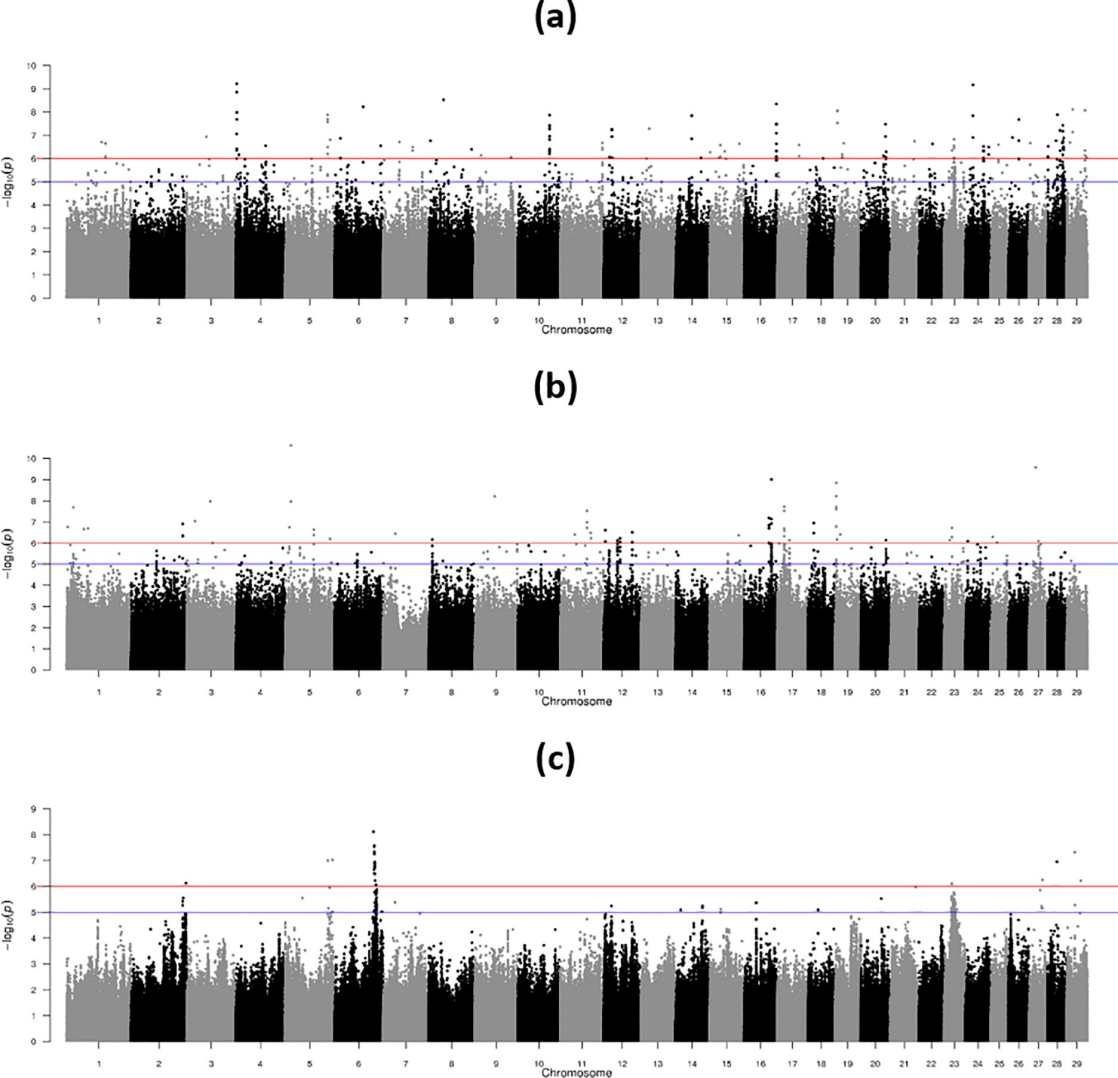
Manhattan plot showing–log_10_ (P-values) of association between each single nucleotide polymorphism effect and the weighted deregressed EBV for bovine tuberculosis infection in the within-breed analyses for (a) the 2,039 purebred Charolais bulls, (b) the 1,964 purebred Limousin bulls, and (c) the 1,502 purebred Holstein-Friesian bulls; red and blue lines represent genome-wide and suggestive thresholds, respectively.

A Manhattan plot outlining the association between each SNP and the EBV for bTB infection for the 2,039 purebred Charolais bulls, the 1,964 purebred Limousin bulls, and the 1,502 purebred Holstein-Friesian bulls are in Fig 6. For the Holstein-Friesians, 62 SNPs were significantly associated with bTB infection at the genome-wide threshold of P = 1 x 10^−6^ (S3 Table); the SNPs that had the strongest association (P ≤ 4.55 x 10^−8^) with bTB infection were all located on BTA6 (genetic position: 96523708 to 98691990). Thirty-nine QTL regions were identified in 15 autosomes for the Holstein-Friesians. Results from the single-SNP regression in the within-breed analyses of the beef breeds had considerably more spurious significant SNP associations, which were predominantly alleles with a low-frequency (S4 and S5 Tables), in comparison to the Holstein-Friesians (S3 Table). For example, in the Limousin breed there were 120 SNPs deemed significantly associated with bTB infection at the genome-wide threshold of P = 1 x 10^−6^ (S4 Table) and 150 QTL regions were defined across all 29 autosomes. For the Charolais breed, there were 325 SNPs deemed significantly associated with bTB infection at the genome-wide threshold of P = 1 x 10^−6^ (S5 Table) and 217 QTL regions were again defined across all 29 autosomes.

A list of the QTL regions from the multi-breed analysis which overlapped with each of the within-breed analyses is in S2 Table. There were just two QTL identified in the multi-breed analysis, both on BTA23, that overlapped with all the within-breed analyses (genetic positions 19441898 to 19592407 and 19617330 to 19662590). As a combined QTL region (i.e., measured from the furthest upstream to the furthest downstream genetic position of the QTL regions in the four analyses), the region was just 0.51 Mb in length (genetic position 19441898 to 19948399) and included six genes namely *RCAN2*, *ENSBTAG00000022736*, *CYP39A1*, *RF00026*, *SLC25A27*, *TDRD6*, and *PLA2G7*. The number of QTL regions from the multi-breed analysis that overlapped with the Charolais, Limousin, and Holstein-Friesian analysis was seven (BTA3, BTA5, BTA6, BTA10, BTA20, and BTA23), nine (BTA2, BTA7, BTA12, BTA13, BTA15, BTA17, and BTA23), and seven (BTA6, BTA7, BTA23, and BTA29), respectively. Forty-six of the 64 QTL regions from the multi-breed analysis did not overlap with any QTL region in either of the within-breed analyses. Of the 38 SNPs in the multi-breed analysis that were deemed to be associated with bTB infection at the genome-wide threshold of P = 1 x 10^−6^, twenty-nine of those SNPs (1 SNP on BTA6, 2 SNPs on BTA10, 23 SNPs on BTA15, and 3 SNPs on BTA16) were not defined in a QTL in any of the within-breed analyses; generally those SNPs were fixed in the Holstein-Friesian population while they were in very low frequency, although segregating, in the Charolais and Limousin populations (S1 Table). For example, 28 of the 29 SNPs were fixed in the Holstein-Friesian bulls, while the 29^th^ SNP (BTA6; genetic position: 45305344) had a P-value of 2.6 x 10^−4^ which was slightly above the cut-off threshold used in the present study for deriving QTLs. Even when a higher MAF threshold (0.01) was applied to the data, there were many QTL in the multi-breed analysis that did not overlap with any QTL from either of the within-breed analysis (S6 Table).

## Discussion

Breeding for resistance to bTB infection in cattle could make a substantial contribution to achieving the national goal of biological extinction of bTB in the Republic of Ireland by 2030 [1]; that is, if exploitable genetic variability among Irish cattle exists. Therefore, the principle objective of the present study was to develop the pertinent phenotype and exposure definition for bTB infection, as well as the editing criteria, statistical methodology, and variance components to undertake routine national genetic evaluations. Moreover, the present study identified a gap in the literature on genome-based association analyses for bTB which have not used whole genome sequence data and they have not considered beef breeds.

### Genetic variability

The genetic standard deviation (0.09) as well as the moderate heritability estimate (0.12) and the repeatability estimate (0.30) for bTB infection in the present study are comparable with previous studies among cattle, even though the phenotype definition and models used sometimes differed [6, 8, 9]. Using 68,497 UK Holstein cows, Brotherstone et al. [8] reported heritability and repeatability estimates on the liability scale for bTB, using four phenotypes, ranging from 0.073 (SE = 0.013) to 0.181 (SE = 0.019) and from 0.159 (SE = 0.013) to 0.329 (SE = 0.013), respectively; heritability estimates from Brotherstone et al. [8] could not be transformed to linear model estimates as the mean incidence of bTB in the dataset was not reported. In an analysis of 184,302 Irish beef and dairy cattle, which were a sub-population of the present study, Richardson et al. [6] reported a genetic standard deviation for bTB of 0.094 units, with a corresponding heritability estimate on the observed scale of 0.11 (SE = 0.006). More recently, Banos et al. [9] also reported variance components for three phenotype definitions of bTB in UK Holsteins; the genetic standard deviation ranged from 0.077 to 0.083 units, heritability estimates on the logit scale ranged from 0.085 (SE = 0.007) to 0.115 (SE = 0.014), and repeatability estimates ranged from 0.697 (SE = 0.002) to 0.699 (SE = 0.005). The higher repeatability estimates documented by Banos et al. [9] compared to the present study are likely due to the interval-model analyses they utilized which allowed each animal to have repeated records within a bTB breakdown; the present study allowed only one record per animal per bTB breakdown.

Results from the present study, supported by previous studies, clearly suggest that genetic selection could play a pivotal role in the eradication and control of bTB (and possibly associated diseases) in cattle populations. For example, if an animal’s EBV for bTB infection were known to producers at the time of selecting parents of the next generation, that EBV is likely to influence mating decisions. By way of example, the dairy AI bull born in 2011 which had the worst (highest) EBV for bTB infection (Fig 4; EBV: 0.18; reliability: 76%) is currently ranked in the best 6% of all dairy animals on the Economic Breeding Index (i.e., the Irish breeding goal for dairy cattle). That bull has 7,260 progeny (source: www.icbf.com); 38% of his progeny in bTB infected herds (29 out 77 progeny across 34 herds) have been diagnosed with bTB infection during their lifetime. If AI companies/producers were aware of his genetic merit for bTB infection at the time of selection it is unlikely he would have been used so frequently.

The potential role of genetic selection in the eradication and control of bTB in cattle populations is further substantiated by the 2.45 percentage unit higher prevalence of positive bTB results in the cohort of high risk EBV animals compared to the cohort of low risk EBV animals when only pedigree relationships were used to predict EBVs; despite the mean bTB EBV reliability being only 19%, considerable observable differences in bTB infection prevalence were observed in the genetically divergent cohorts. If the mean bTB EBV reliability were improved through, for example, targeted genome-based predictions of genetic merit, a greater differentiation in bTB infection prevalence could be observed among the genetically divergent groups.

The consequences of preventing just one bTB infection in a 100-cow herd, assuming an infected animal is also infectious, could avert a subsequent 4.9 secondary bTB infections (reproductive ratio of 4.9) [30] arising from cattle-to-cattle transmission of bTB. Woolhouse et al. [31] estimated that just 20% of cattle herds are responsible for transmitting 80% of infectious diseases; therefore, if the use of genetic selection for resistance to bTB was targeted towards herds or geographical locations with a history of high bTB infection, the goal of eradication [1] could be realized by 2030. Interestingly, the Irish Cattle Breeding Federation (ICBF) in the Republic of Ireland is very unique in that the organization comprises information from many sources (e.g., veterinarians, abattoirs, and milk-processors) in one central database to provide producers with reports and breeding services that can be tailored to the specific requirements of each animal and herd. One such ‘mating-advice’ service provided by ICBF aims to optimize cow and bull matings (based on genetic merit for economically important traits) to generate offspring that should, on average, be more profitable. This facility could potential identify herds in geographical locations that are at-risk of bTB, using bTB phenotypes, and tailor the mating-advice in these herds accordingly to favor bull-dam matings with bTB resistant bulls.

The implications of including bTB in Irish dairy and beef breeding goals is unlikely to have strong negative ramifications on other traits in each breeding goal given the lack of any consistent antagonistic genetic correlation between bTB and performance traits [32]. This view is supported by the favorable annual genetic trend on bTB for dairy AI sires which suggests that the existing dairy breeding goal is already breeding for resistance to bTB infection, even though bTB is not currently explicitly included in the breeding goal. Nonetheless, 8% (beef) and 11% (dairy) of the relative emphasis in the existing breeding goals in the Republic of Ireland are comprised of a survival trait which selects cows that are genetically more likely to survive longer in the herd. Survival is often incorrectly considered as only a fertility trait, given its strong correlation with calving interval [33–34] and the phenotype used to derive the trait (consecutive calving events). Nonetheless, 38% of the variability in survival can be attributed to responsiveness to bTB tests (assuming a genetic correlation between survival and bTB of -0.62) [32]. Therefore, genetic selection for improved survival alone, irrespective of whether bTB is explicitly included in Irish breeding goals, should continue to reduce the prevalence of bTB in both Irish dairy and beef cattle. That said, if bTB was included in Irish breeding goals, a faster rate of genetic gain could be achieved. For example, in a single-trait section index where the goal standard trait is cow survival (100 progeny records available and an economic weight €12.43) [35] and no records are available for bTB, with each 0.1 percentage unit improvement in cow survival, the prevalence of bTB should reduce by 0.09 percentage units; this scenario assumes genetic parameters for survival and bTB were equivalent to Ring et al. [36] and the present study, respectively, and the genetic correlation between survival and bTB was -0.62 [32]. In a subsequent scenario, where survival remains the goal standard trait, but 100 progeny records of bTB were also available to complement the existing 100 survival records, with each 0.1 percentage unit improvement in cow survival, the rate at which bTB prevalence is reduced should double compared to when no bTB records are available.

### Genome-wide association

It is assumed that bTB is a complex polygenic trait [10]; therefore many genetic variants, each with a small effect, are likely to regulate the observed phenotype. The extent to which multi-breed genetic variants influence resistance to bTB is unclear as, to-date, all genome-wide association analyses for resistance to bTB on *Bos taurus* cattle have been restricted to just the Holstein-Friesian breed [5–6, 10–12]. The benefits of utilizing results from genome-wide association analyses which have been restricted to just the Holstein-Friesian breed may not fully inform the biological process underlying bTB infection in all *Bos taurus* breeds. The use of multi-breed genome-wide association analyses has been investigated previously for milk production [37] and disease traits [38]. Van den Berg et al. [37] reported an increase in the statistical power of detecting variants associated with milk production using imputed whole genome sequence data when using a multi-breed genome-wide association analysis compared to a within-breed genome-wide association analysis, especially when dairy populations were closely related; moreover, when the dairy populations were distantly related, the mapping precision of the genome-wide association analysis improved when multi-breed genome-wide association analysis were used [37]. Following within-breed genome-wide association analysis studies on *M*. *paratuberculosis* [39–41], Sallam et al. [38] preformed a multi-breed genome-wide association on the same *M*. *paratuberculosis* population and phenotype. Sallam et al. [38] observed many significant SNP associations in the multi-breed analysis which had not been detected in the within-breed analyses. While the present study noted some overlap in the QTL regions associated with TB in the multi-breed analysis compared to the within-breed analyses, many different QTL regions for the same trait were identified across the four different analyses.

#### Positional candidate regions

The present study identified several regions of the bovine genome associated with bTB infection, but none of the SNPs or QTL regions in the multi-breed analysis overlapped with SNPs previously reported for bTB infection; in comparison to the previous five genome-based association studies on bTB infection, each of those studies only considered Holstein-Friesian cattle and they were limited by sample size (range: 307 to 1151 cattle) [5–6, 10–12]. Even when the single-SNP regression analysis in the present study was reduced from the multi-breed analysis of 7,346 bulls to just the 1,502 Holstein-Friesian bulls, no significant SNP association agreed with those previously documented, and no SNP identified in previous studies for resistance to bTB was in a QTL defined in the present study. For example, based on analyses of 841 Holstein-Friesian bulls, Richardson et al. [7] reported a SNP at the genetic position of 9591806 on BTA23 as the most suggestive of bTB infection. The T allele that Richardson et al. [7] suggested was favorable for resistance to bTB was also favorable for the Holstein-Friesian bulls in the present study (frequency of T allele in Holstein-Friesians: 0.147), but not in the multi-breed analysis of 7,346 bulls, while the SNP itself was far from significant (P-value when analysis was restricted to Holstein-Friesians: 0.135). Based on the within-breed analysis of Holstein-Friesian bulls in the present study, there was no SNP with a P-value < 1 x 10^−6^ that flanked 1Mb of the SNP deemed most suggestive of bTB resistance by Richardson et al. [7]; the closest SNP with a P-value < 1 x 10^−6^ was 9.85 Mb downstream.

Using 803 Holstein-Friesian sires, Raphaka et al. [10] reported a 0.57 allele substitution effect for a SNP with a genetic position of 93065483 on BTA2 where the minor allele (frequency: 0.37) was associated with increased resistance to bTB; the present study also noted an allele substitution effect of the same direction for the minor G allele (frequency: 0.34) within Holstein-Friesian bulls, albeit of lesser magnitude (0.005; P = 0.053). Based on the within-breed analysis of Holstein-Friesian bulls in the present study, the nearest QTL region defined on BTA2 was 33.7 Mb downstream of the SNP reported as significant by Raphaka et al. [10]. Interestingly, Raphaka et al. [10] defined a large QTL region (8.18 Mb) on BTA23 (genetic position: 30222836 to 38412668) for two (of three) bTB phenotypes investigated and that QTL region did overlap with one smaller region (26 Kb) in the present study (genetic position: 32333875 to 32359891) when only Holstein-Friesians were considered.

In comparison to three other genome-wide associations on bTB among Holstein-Friesian cattle which documented SNPs of interest on BTA2 (genetic position: 25899036; [5]), BTA6 (genetic position: 10245091; [11]), BTA13 (genetic positions: 71782488, 71784332, 71787722, 71788784, 71789620, and 71791844; [5]), and BTA22 (genetic positions: 58557374, 58530759, and 58597169; [12]), those SNPs were not deemed to be significantly associated with bTB infection in the present study in either the multi-breed analysis (P values ranged from 0.16 to 0.96) or in the within-breed analysis of Holstein-Friesians (P values ranged from 0.18 to 0.96); nonetheless, the frequency of the favorable allele for those SNPs in the Holstein-Friesians was moderately high, with the exception of one SNP (BTA2; genetic position 25899036) reported by Bermingham et al. [5]. Where SNPs were identified on the same autosome as other studies, the QTLs defined in the within-breed analysis of Holstein-Friesians in the present study were at least 86 Mb upstream or downstream of the most significant SNPs reported by Finlay et al. [12], Bermingham et al. [5], and Tsairidou et al. [11].

#### Functional candidate gene

In mice, the *NRAMP1* gene has sometimes been shown to confer resistance to tuberculosis [42]; however, the links between *NRAMP1* and bTB in cattle are inconsistent [43–44]. In the present study, 18 SNPs in the multi-breed analysis were located in the *NRAMP1* gene, where the SNP with the lowest P-value (P = 0.049; BTA2, genetic position: 107118274) had an allele substitution effect of 0.014, with the major T allele (frequency in the 7,346 bulls: 0.991) being associated with increased resistance to bTB; the unfavorable minor C allele was mostly present as a heterozygous copy in the present study (125 TC bulls; 3 CC bulls). For that SNP, the within-breed frequency of the favorable allele, which was T for all breeds, was 0.983 for Belgian Blue, 0.998 for Charolais, 0.986 for Hereford, 0.980 for Holstein-Friesian, 0.993 for Limousin, and 0.994 for Simmental; the favorable T allele was fixed in the Aberdeen Angus breed.

### Olfactory regions may signal avoidance of bTB in pasture-based production systems

Medzhitov et al. [45] documented three distinct techniques that hosts could potentially employ to protect oneself from disease; these include avoidance, resistance, and tolerance. The avoidance strategy was highlighted by Phillips et al. [46] who suggested that grazing habits or social structure among cattle, with respect to avoidance of badger excreta, may genetically influence resistance to bTB infection. The avoidance strategy requires detection of the pathogen, or perhaps the location of the pathogen (e.g., feces), by the host prior to infection, and subsequent alteration of the host’s behavior; detection of pathogens (or the location of pathogens) is facilitated primarily by olfactory and gustatory systems [45]. Despite the frequency of alleles being very low (< 0.01), the molecular function of four of the total eleven genes identified in SNPs that surpassed the genome-wide threshold in the multi-breed analysis in the present study have a molecular function in olfactory receptor activity; the olfactory receptor activity is responsible for the transmission of signals and the initiation of change in cell activity, in response to odor detection. In support of this hypothesis, Smith et al. [47] conducted a grazing behavioral study in the UK in which feces from multiple species contaminated pasture swards prior to cattle grazing. Smith et al. [47] reported that cattle were least likely to graze badger-contaminated swards, compared to any sward contaminated by other species. Notably, badgers (*Meles meles*) are deemed to be the primary wildlife reservoir of bTB in the Republic of Ireland [48] and they are known to transmit the disease to cattle [49], yet badgers tend to avoid cattle in pasture as well as cattle housing [50–53], thus direct contact between badgers and cattle is rare. Moreover, in the study by Smith et al. [47], when cattle were offered only badger-contaminated swards of varying quantities of contamination, cattle consumed the swards that were most densely contaminated the least. Our results, coupled with the results from the behavioral study by Smith et al. [47] and others [50–53], suggest that at least in pasture-based production systems breeding for the favorable minor allele of SNPs in the olfactory receptor genes on BTA15 may reduce the likelihood of cattle becoming infected with bTB as a result of fewer cattle consuming badger contaminated pasture. Results from the present study should, however, be interpreted with caution due to the very low frequency of favorable alleles in the population. While the favorable alleles of SNPs in the olfactory genes were most abundant in the Hereford bulls (S1 Table), the potential of breeding for the favorable allele is high given that most significant SNPs were segregating in each breed, except Holstein-Friesians; that said, Hereford is one of the mostly widely-used beef breeds to sire calves in Irish dairy herds (http://www.agriculture.gov.ie) thus, offspring from Holstein-Friesians that are more resistant to bTB could be generated by mating Herefords that have the favorable allele with Holstein-Friesians. It could also be hypothesized that the Hereford breed which originated in the British Isles may have acquired improved resistance to bTB during its extensive time-period of exposure to bTB.

### Role of BTA23 in resistance to bTB infection

Although agreement has not been reached on the specific region(s) of interest for resistance to bTB, previous studies have repeatedly highlighted the association between resistance to bTB infection and BTA23 [7, 10]; the present study supports these results. Each of the significant SNPs identified on BTA23 in the multi-breed analysis were segregating in all breeds and the frequency of the favorable allele was moderately high (range: 0.593 to 0.988; S1 Table), indicating genetic selection could amplify the frequency of the favorable allele in each breed. In the present study, three QTLs on BTA23, spanning from 19.441 to 22.727 Mb, were defined in the multi-breed analysis (S2 Table) while a further 9 QTLs in the Charolais, 7 QTLs in the Limousin, and 12 QTLs in the Holstein-Friesians were again identified on BTA23. Interestingly, there was only one QTL region (genetic position 19441898 to 19948399) that overlapped among the four analyses in the present study, and that QTL was located on BTA23; that region included six genes, including *RCAN2*, *ENSBTAG00000022736*, *CYP39A1*, *RF00026*, *SLC25A27*, *TDRD6*, and *PLA2G7*. Notably, 11 SNPS with P-values ≤ 1.47 x 10^−4^ were upstream gene variants of *RCAN2* while another SNP with a P-value of 0.05 was classified as a missense gene variant. Interestingly, *RCAN2* is responsible for the regulation of calcineurin 2 and calcineurin activation promotes the survival of tuberculosis within its host by preventing phagocyte maturation (which is required to destroy tuberculosis; [54]).

## Conclusions

Breeding for resistance to bTB is a plausible strategy complementing existing approaches that aim to eradicate bTB from the Republic of Ireland (and elsewhere). Results from the present study substantiate that considerable exploitable genetic variability exists in resistance to bTB. Moreover, SNPs putatively associated with bTB infection have been identified which have not been previously linked to bTB. While results from the present study should be interpreted, our results, coupled with previous studies, suggest that olfactory receptor genes as well as regulation of calcineurin 2 may have a pivotal role in the resistance of cattle to bTB. Methods and results from the present study can be used to develop national genetic evaluations for bTB in the Republic of Ireland and elsewhere. In addition, results can be used to help uncover the biological architecture underlying resistance to bTB infection in cattle.

## Supporting information

S1 Fig QQ-plot of observed–log10 (P-values) versus expected–log10 (P-values) in the multi-breed analysis. (TIFF)

S1 Table Chromosome (BTA), position, favorable allele, and the frequency of the favorable allele for the breeds most represented in the dataset for each of the 38 single nucleotide polymorphisms that were associated with bovine tuberculosis infection in the multi-breed analysis (P < 1 x 10^−6^). (DOCX)

S2 Table Chromosome (BTA), start position, and end position of each quantitative trait loci region defined in the multi-breed analysis of 7,346 bulls as well as the quantitative trait loci that overlapped in the within-breed analysis for the 2,039 purebred Charolais bulls (CH), the 1,964 purebred Limousin bulls (LM), and the 1,502 purebred Holstein-Friesian bulls (HO). (DOCX)

S3 Table Chromosome (BTA), position, P-value, the favorable allele, the frequency of the favorable allele, substitution effect of the favorable allele, annotation, and gene for the 62 single nucleotide polymorphisms associated with bovine tuberculosis infection in the within-breed analysis of Holstein-Friesian bulls (P < 1 x 10^−6^). (DOCX)

S4 Table Chromosome (BTA), position, P-value, the favorable allele, the frequency of the favorable allele, substitution effect of the favorable allele, annotation, and gene for the 120 single nucleotide polymorphisms associated with bovine tuberculosis infection in the within-breed analysis of Limousin bulls (P < 1 x 10^−6^). (DOCX)

S5 Table Chromosome (BTA), position, P-value, the favorable allele, the frequency of the favorable allele, substitution effect of the favorable allele, annotation, and gene for the 325 single nucleotide polymorphisms associated with bovine tuberculosis infection in the within-breed analysis of Charolais bulls (P < 1 x 10^−6^). (DOCX)

S6 Table Chromosome (BTA), start position, and end position of each quantitative trait loci region defined in the multi-breed analysis (All) and the within-breed analysis of Charolais bulls (CH), Limousin bulls (LM), or Holstein-Friesian bulls (HO) as well as the quantitative trait loci that overlapped between analyses when quantitative trait loci were triggered by SNPs with P-value < 1 x 10^−5^ that also had a minor-allele frequency of >0.01. (DOCX)
