# Supplementary figures and images for: Variance components for bovine tuberculosis infection and multi-breed genome-wide association analysis using imputed whole genome sequence data
Source: PLoS One. 2019 Feb 14;14(2):e0212067. doi: 10.1371/journal.pone.0212067 (PMC6375599; doi:10.1371/journal.pone.0212067)

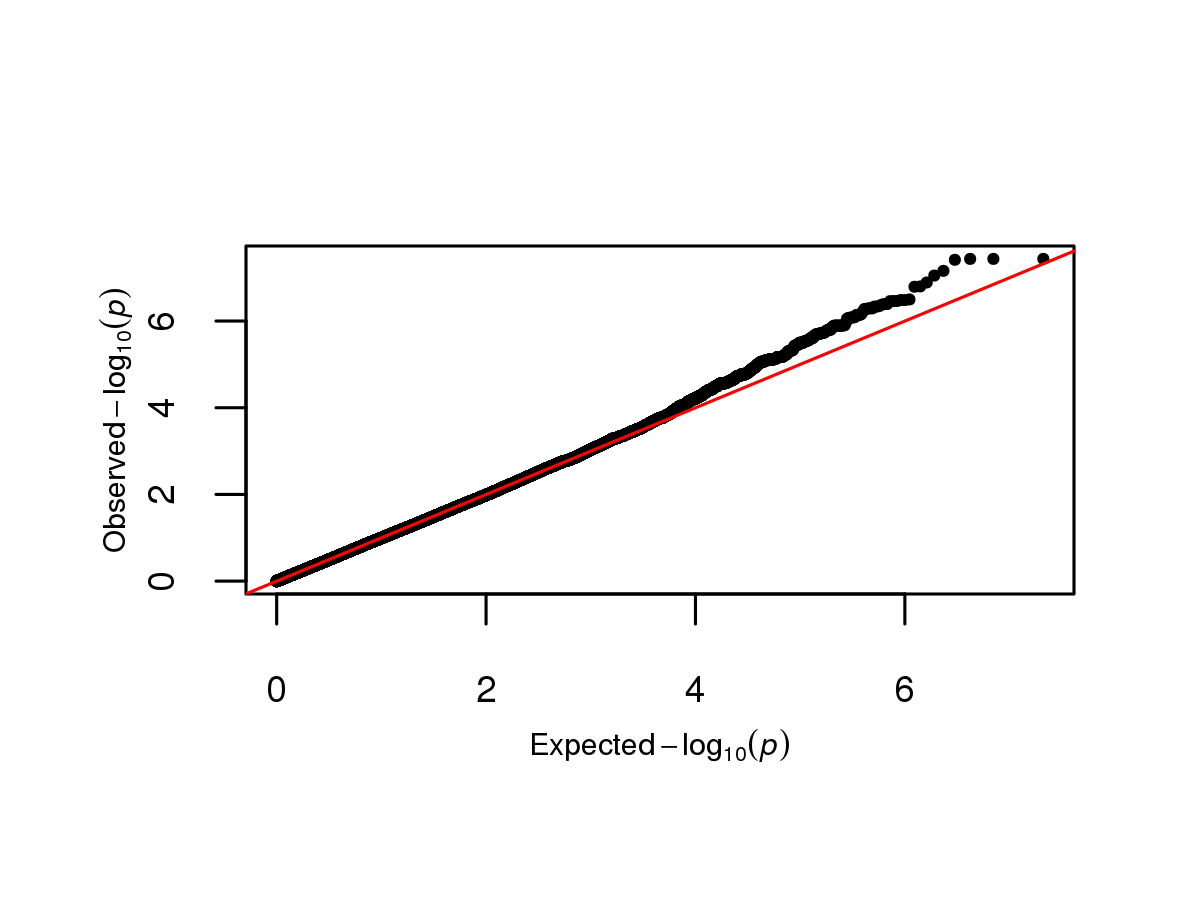

Supplement: S1 Fig — (TIFF) [file pone.0212067.s001.tiff]
