# Supplementary material for: Variance components for bovine tuberculosis infection and multi-breed genome-wide association analysis using imputed whole genome sequence data
Source: PLoS One. 2019 Feb 14;14(2):e0212067. doi: 10.1371/journal.pone.0212067 (PMC6375599; doi:10.1371/journal.pone.0212067)
Supplement: S1 Table — (DOCX) [file pone.0212067.s002.docx]

**Table S1.** Chromosome (BTA), position, favorable allele, and the frequency of the favorable allele for the breeds most represented in the dataset for each of the 38 single nucleotide polymorphisms that were associated with bovine tuberculosis infection in the multi-breed analysis (P < 1 x 10^-6^)

| BTA | Position | Allele | Aberdeen Angus | Belgian Blue | Charolais | Hereford | Holstein-Friesian | Limousin | Simmental |
| --- | --- | --- | --- | --- | --- | --- | --- | --- | --- |
| 6 | 45305344 | T | 0.997 | 1.000 | 0.996 | 1.000 | 0.990 | 0.992 | 0.997 |
| 6 ^a^ | 96523708 | G | 1.000 | 1.000 | 0.999 | 1.000 | 0.981 | 1.000 | 1.000 |
| 6 ^a^ | 96544980 | G | 1.000 | 1.000 | 0.999 | 1.000 | 0.981 | 1.000 | 1.000 |
| 10 | 10238600 | G | 0.919 | 1.000 | 0.998 | 1.000 | 1.000 | 0.998 | 1.000 |
| 10 | 10268287 | C | 0.919 | 1.000 | 0.998 | 1.000 | 1.000 | 0.998 | 1.000 |
| 15 | 47655527 | C | 0.001 | 0.002 | 0.002 | 0.103 | 0.000 | 0.002 | 0.002 |
| 15 | 47657277 | T | 0.001 | 0.002 | 0.002 | 0.103 | 0.000 | 0.002 | 0.002 |
| 15 | 47671329 | A | 0.001 | 0.002 | 0.002 | 0.103 | 0.000 | 0.002 | 0.002 |
| 15 | 47672311 | T | 0.001 | 0.002 | 0.002 | 0.103 | 0.000 | 0.002 | 0.002 |
| 15 | 47682180 | G | 0.001 | 0.002 | 0.001 | 0.103 | 0.000 | 0.002 | 0.001 |
| 15 | 47682642 | A | 0.001 | 0.002 | 0.001 | 0.103 | 0.000 | 0.002 | 0.001 |
| 15 | 47683747 | A | 0.001 | 0.002 | 0.002 | 0.103 | 0.000 | 0.002 | 0.002 |
| 15 | 47688637 | C | 0.001 | 0.010 | 0.002 | 0.103 | 0.000 | 0.002 | 0.002 |
| 15 | 47695516 | T | 0.001 | 0.007 | 0.001 | 0.105 | 0.000 | 0.002 | 0.002 |
| 15 | 47729320 | T | 0.001 | 0.000 | 0.001 | 0.101 | 0.000 | 0.001 | 0.003 |
| 15 | 47735420 | A | 0.001 | 0.007 | 0.001 | 0.103 | 0.000 | 0.001 | 0.005 |
| 15 | 47738084 | A | 0.001 | 0.000 | 0.000 | 0.101 | 0.000 | 0.001 | 0.003 |
| 15 | 47740797 | T | 0.001 | 0.000 | 0.000 | 0.101 | 0.000 | 0.001 | 0.003 |
| 15 | 47750477 | A | 0.001 | 0.000 | 0.001 | 0.101 | 0.000 | 0.001 | 0.003 |
| 15 | 47751834 | C | 0.001 | 0.000 | 0.001 | 0.101 | 0.000 | 0.001 | 0.003 |
| 15 | 47752145 | C | 0.001 | 0.000 | 0.003 | 0.101 | 0.000 | 0.001 | 0.003 |
| 15 | 47754771 | A | 0.001 | 0.000 | 0.003 | 0.101 | 0.000 | 0.001 | 0.003 |
| 15 | 47755338 | A | 0.001 | 0.000 | 0.003 | 0.101 | 0.000 | 0.001 | 0.003 |
| 15 | 47765272 | A | 0.001 | 0.000 | 0.003 | 0.101 | 0.000 | 0.001 | 0.003 |
| 15 | 48067616 | T | 0.003 | 0.000 | 0.001 | 0.098 | 0.000 | 0.000 | 0.001 |
| 15 | 48070477 | G | 0.003 | 0.000 | 0.001 | 0.098 | 0.000 | 0.000 | 0.001 |
| 15 | 48075512 | G | 0.003 | 0.000 | 0.001 | 0.098 | 0.000 | 0.000 | 0.001 |
| 15 | 48159563 | T | 0.004 | 0.000 | 0.002 | 0.098 | 0.000 | 0.001 | 0.003 |
| 16 | 4041643 | G | 0.995 | 0.998 | 0.965 | 1.000 | 1.000 | 0.987 | 0.995 |
| 16 | 4080552 | A | 0.994 | 0.995 | 0.964 | 0.998 | 1.000 | 0.986 | 0.994 |
| 16 | 4168671 | G | 0.999 | 0.995 | 0.964 | 1.000 | 1.000 | 0.987 | 0.995 |
| 17 ^a^ | 17595339 | A | 1.000 | 1.000 | 0.999 | 1.000 | 1.000 | 0.991 | 1.000 |
| 23 ^a^ | 19465559 | C | 0.759 | 0.593 | 0.761 | 0.787 | 0.771 | 0.776 | 0.808 |
| 23 ^a^ | 19632067 | G | 0.910 | 0.706 | 0.915 | 0.988 | 0.869 | 0.856 | 0.945 |
| 23 ^a^ | 19634225 | C | 0.910 | 0.706 | 0.915 | 0.988 | 0.869 | 0.856 | 0.945 |
| 23 ^a^ | 19641604 | G | 0.908 | 0.699 | 0.908 | 0.935 | 0.839 | 0.837 | 0.942 |
| 23 ^a^ | 19642280 | G | 0.910 | 0.706 | 0.915 | 0.988 | 0.869 | 0.855 | 0.945 |
| 23 ^a^ | 19657604 | C | 0.910 | 0.699 | 0.915 | 0.988 | 0.868 | 0.855 | 0.945 |

^a^ Single nucleotide polymorphism was identified in a quantitative trait loci region in the within-breed analyses of either the 2,039 purebred Charolais bulls, the 1,964 purebred Limousin bulls, or the 1,502 purebred Holstein-Friesian bulls
