# Supplementary material for: Variance components for bovine tuberculosis infection and multi-breed genome-wide association analysis using imputed whole genome sequence data
Source: PLoS One. 2019 Feb 14;14(2):e0212067. doi: 10.1371/journal.pone.0212067 (PMC6375599; doi:10.1371/journal.pone.0212067)
Supplement: S2 Table — (DOCX) [file pone.0212067.s003.docx]

**Table S2.** Chromosome (BTA), start position, and end position of each quantitative trait loci region defined in the multi-breed analysis of 7,346 bulls as well as the quantitative trait loci that overlapped in the within-breed analysis for the 2,039 purebred Charolais bulls (CH), the 1,964 purebred Limousin bulls (LM), and the 1,502 purebred Holstein-Friesian bulls (HO)

| BTA | Start | End | Overlapping  within-breed |  | BTA | Start | End | Overlapping  within-breed |
| --- | --- | --- | --- | --- | --- | --- | --- | --- |
| 1 | 28,005,508 | 28,005,508 |  |  | 15 | 44,461,212 | 46,371,067 | LM |
| 1 | 71,746,607 | 71,761,506 |  |  | 15 | 47,655,527 | 47,765,272 |  |
| 2 | 20,073,825 | 20,073,825 |  |  | 15 | 48,067,616 | 48,400,758 |  |
| 2 | 62,187,019 | 62,778,428 | LM |  | 16 | 4,041,643 | 4,195,105 |  |
| 3 | 49,207,894 | 49,570,924 |  |  | 16 | 9,896,461 | 9,896,461 |  |
| 3 | 68,660,720 | 68,754,319 |  |  | 16 | 63,052,720 | 63,288,669 |  |
| 3 | 88,471,915 | 90,292,003 | CH |  | 16 | 66,385,630 | 66,385,630 |  |
| 4 | 112,052,508 | 113,050,432 |  |  | 17 | 15,120,437 | 20,347,952 | LM |
| 5 | 51,908,357 | 52,728,346 |  |  | 17 | 22,963,268 | 23,484,201 | LM |
| 5 | 62,550,865 | 68,368,842 | CH |  | 17 | 53,109,293 | 53,319,505 |  |
| 6 | 44,998,290 | 45,473,675 |  |  | 17 | 66,216,719 | 66,228,330 |  |
| 6 | 45,866,075 | 45,943,809 |  |  | 18 | 19,339,668 | 19,349,400 |  |
| 6 | 61,226,945 | 62,889,291 |  |  | 19 | 13,215,245 | 13,215,245 |  |
| 6 | 96,382,367 | 96,621,131 | HO |  | 19 | 42,168,012 | 42,327,305 |  |
| 6 | 102,640,308 | 102,691,067 | HO |  | 20 | 2,366,300 | 2,401,163 |  |
| 6 | 116,712,958 | 116,712,958 | CH |  | 20 | 29,023,272 | 29,023,272 |  |
| 7 | 30,655,582 | 31,251,154 | HO; LM |  | 20 | 60,858,032 | 60,965,117 | CH |
| 8 | 3,018,432 | 3,033,135 |  |  | 22 | 53,919,225 | 54,174,356 |  |
| 8 | 23,897,013 | 24,134,049 |  |  | 22 | 54,222,900 | 54,305,381 |  |
| 9 | 72,193,061 | 72,247,962 |  |  | 22 | 54,406,663 | 54,406,663 |  |
| 9 | 72,293,978 | 72,317,521 |  |  | 22 | 54,502,459 | 54,716,005 |  |
| 9 | 89,459,738 | 89,469,653 |  |  | 23 | 19,441,898 | 19,592,407 | CH; HO; LM |
| 10 | 10,001,469 | 10,363,751 |  |  | 23 | 19,617,330 | 19,662,590 | CH; HO; LM |
| 10 | 10,489,558 | 10,599,973 |  |  | 23 | 22,718,870 | 22,727,379 | HO |
| 10 | 66,935,959 | 66,935,959 | CH |  | 24 | 9,655,417 | 9,659,989 |  |
| 10 | 92,007,484 | 92,028,789 |  |  | 24 | 37,925,240 | 37,925,240 |  |
| 11 | 39,842,572 | 39,891,991 |  |  | 26 | 8,476,143 | 8,476,151 |  |
| 12 | 70,248,991 | 70,271,532 | LM |  | 26 | 41,344,615 | 41,344,680 |  |
| 13 | 35,848,759 | 41,746,215 | LM |  | 27 | 30,187,171 | 30,278,015 |  |
| 13 | 50,693,621 | 51,235,422 |  |  | 29 | 11,750,719 | 11,758,439 |  |
| 14 | 51,632,521 | 51,652,718 |  |  | 29 | 19,166,950 | 19,533,465 |  |
| 15 | 14,595,633 | 14,595,633 |  |  | 29 | 20,687,473 | 21,035,866 | HO |
