# Supplementary material for: Variance components for bovine tuberculosis infection and multi-breed genome-wide association analysis using imputed whole genome sequence data
Source: PLoS One. 2019 Feb 14;14(2):e0212067. doi: 10.1371/journal.pone.0212067 (PMC6375599; doi:10.1371/journal.pone.0212067)
Supplement: S3 Table — (DOCX) [file pone.0212067.s004.docx]

**Table S3.** Chromosome (BTA), position, P-value, the favorable allele, the frequency of the favorable allele, substitution effect of the favorable allele, annotation, and gene for the 62 single nucleotide polymorphisms associated with bovine tuberculosis infection in the within-breed analysis of Holstein-Friesian bulls (P < 1 x 10^-6^)

| BTA | Position | P-value | Allele | Frequency | Effect | Annotation | Gene |
| --- | --- | --- | --- | --- | --- | --- | --- |
| 2 | 135023310 | 7.51x10^-7^ | T | 0.997 | 0.126 | intergenic |  |
| 2 | 135023810 | 7.51x10^-7^ | C | 0.997 | 0.126 | intergenic |  |
| 5 | 105336901 | 1.00x10^-7^ | A | 0.998 | 0.152 | intergenic |  |
| 5 | 105366936 | 1.00x10^-7^ | A | 0.998 | 0.152 | intergenic |  |
| 5 | 116507241 | 9.44x10^-8^ | G | 0.994 | 0.093 | intron | SMC1B |
| 6 | 96523708 | 7.71x10^-9^ | G | 0.981 | 0.053 | intron | ANTXR2 |
| 6 | 96544980 | 7.71x10^-9^ | G | 0.981 | 0.053 | intergenic |  |
| 6 | 96580339 | 7.71x10^-9^ | T | 0.981 | 0.053 | intergenic |  |
| 6 | 97680389 | 2.25x10^-7^ | A | 0.946 | 0.028 | intron | PRKG2 |
| 6 | 98174672 | 2.91x10^-8^ | A | 0.976 | 0.046 | intergenic |  |
| 6 | 98500120 | 2.68x10^-8^ | G | 0.976 | 0.047 | intergenic |  |
| 6 | 98502075 | 2.68x10^-8^ | C | 0.976 | 0.047 | intergenic |  |
| 6 | 98509366 | 2.68x10^-8^ | A | 0.976 | 0.047 | intergenic |  |
| 6 | 98515316 | 5.55x10^-8^ | G | 0.975 | 0.045 | intergenic |  |
| 6 | 98516620 | 5.55x10^-8^ | T | 0.975 | 0.045 | intergenic |  |
| 6 | 98551893 | 5.55x10^-8^ | C | 0.975 | 0.045 | intergenic |  |
| 6 | 98552832 | 5.55x10^-8^ | C | 0.975 | 0.045 | intergenic |  |
| 6 | 98571734 | 3.14x10^-7^ | C | 0.973 | 0.040 | intergenic |  |
| 6 | 98691990 | 4.56x10^-8^ | G | 0.976 | 0.046 | intergenic |  |
| 6 | 99293468 | 1.30x10^-7^ | C | 0.974 | 0.043 | intron | SCD5 |
| 6 | 99405866 | 1.73x10^-7^ | C | 0.972 | 0.041 | intron | SCD5 |
| 6 | 99410743 | 1.15x10^-7^ | G | 0.974 | 0.043 | 5' UTR variant | SCD5 |
| 6 | 99412045 | 1.15x10^-7^ | T | 0.974 | 0.043 | upstream gene | SCD5 |
| 6 | 99412575 | 1.15x10^-7^ | G | 0.974 | 0.043 | upstream gene | SCD5 |
| 6 | 99415740 | 1.15x10^-7^ | A | 0.974 | 0.043 | upstream gene | SCD5 |
| 6 | 99428084 | 1.15x10^-7^ | C | 0.974 | 0.043 | intron | SEC31A |
| 6 | 99447136 | 1.61x10^-7^ | C | 0.975 | 0.043 | intron | SEC31A |
| 6 | 99468561 | 1.82x10^-7^ | C | 0.975 | 0.043 | intron | SEC31A |
| 6 | 99469544 | 1.82x10^-7^ | G | 0.975 | 0.043 | intron | SEC31A |
| 6 | 99480905 | 1.82x10^-7^ | T | 0.975 | 0.043 | upstream gene | SEC31A |
| 6 | 99481032 | 1.82x10^-7^ | G | 0.975 | 0.043 | upstream gene | SEC31A |
| 6 | 99489604 | 1.82x10^-7^ | C | 0.975 | 0.043 | intergenic |  |
| 6 | 99496757 | 1.82x10^-7^ | C | 0.975 | 0.043 | upstream gene | THAP9 |
| 6 | 99524607 | 3.27x10^-7^ | A | 0.975 | 0.042 | downstream gene | LIN54 |
| 6 | 99550694 | 1.38x10^-7^ | A | 0.975 | 0.044 | intron | LIN54 |
| 6 | 99555722 | 1.38x10^-7^ | A | 0.975 | 0.044 | intron | LIN54 |
| 6 | 99556707 | 1.38x10^-7^ | C | 0.975 | 0.044 | intron | LIN54 |
| 6 | 99557576 | 1.38x10^-7^ | T | 0.975 | 0.044 | intron | LIN54 |
| 6 | 99557677 | 1.38x10^-7^ | C | 0.975 | 0.044 | intron | LIN54 |
| 6 | 99558254 | 1.38x10^-7^ | G | 0.975 | 0.044 | intron | LIN54 |
| 6 | 99563720 | 1.38x10^-7^ | G | 0.975 | 0.044 | intron | LIN54 |
| 6 | 99572981 | 1.38x10^-7^ | A | 0.975 | 0.044 | intron | LIN54 |
| 6 | 99572993 | 1.38x10^-7^ | A | 0.975 | 0.044 | intron | LIN54 |
| 6 | 99586187 | 1.38x10^-7^ | G | 0.975 | 0.044 | downstream gene | bta-mir-2447 |
| 6 | 99586910 | 1.38x10^-7^ | A | 0.975 | 0.044 | downstream gene | bta-mir-2447 |
| 6 | 99589219 | 1.38x10^-7^ | G | 0.975 | 0.044 | upstream gene | bta-mir-2447 |
| 6 | 99593756 | 1.38x10^-7^ | G | 0.975 | 0.044 | intergenic |  |
| 6 | 99614117 | 1.38x10^-7^ | A | 0.975 | 0.044 | intron | COPS4 |
| 6 | 99631553 | 1.38x10^-7^ | G | 0.975 | 0.044 | intron | COPS4 |
| 6 | 99694381 | 1.84x10^-7^ | A | 0.975 | 0.043 | upstream gene | PLAC8 |
| 6 | 99711520 | 1.84x10^-7^ | A | 0.975 | 0.043 | intergenic |  |
| 6 | 99958637 | 1.84x10^-7^ | G | 0.975 | 0.043 | intergenic |  |
| 6 | 100239341 | 5.91x10^-7^ | G | 0.975 | 0.041 | intergenic |  |
| 6 | 102667052 | 8.67x10^-7^ | G | 0.962 | 0.033 | downstream gene | ARHGAP24 |
| 6 | 102667293 | 8.67x10^-7^ | C | 0.962 | 0.033 | downstream gene | ARHGAP24 |
| 23 | 19445247 | 7.85x10^-7^ | A | 0.831 | 0.017 | intron | RCAN2 |
| 23 | 19456307 | 7.85x10^-7^ | C | 0.831 | 0.017 | intron | RCAN2 |
| 27 | 32740386 | 5.64x10^-7^ | G | 0.996 | 0.104 | downstream gene | ERLIN2 |
| 28 | 22910324 | 1.11x10^-7^ | C | 0.998 | 0.155 | intron | CTNNA3 |
| 28 | 22910330 | 1.11x10^-7^ | C | 0.998 | 0.155 | intron | CTNNA3 |
| 29 | 20887648 | 4.79x10^-7^ | G | 0.998 | 0.146 | intergenic |  |
| 29 | 35572213 | 6.02x10^-7^ | G | 0.997 | 0.159 | intron | NTM |
