# Supplementary material for: Variance components for bovine tuberculosis infection and multi-breed genome-wide association analysis using imputed whole genome sequence data
Source: PLoS One. 2019 Feb 14;14(2):e0212067. doi: 10.1371/journal.pone.0212067 (PMC6375599; doi:10.1371/journal.pone.0212067)
Supplement: S4 Table — (DOCX) [file pone.0212067.s005.docx]

**Table S4.** Chromosome (BTA), position, P-value, the favorable allele, the frequency of the favorable allele, substitution effect of the favorable allele, annotation, and gene for the 120 single nucleotide polymorphisms associated with bovine tuberculosis infection in the within-breed analysis of Limousin bulls (P < 1 x 10^-6^)

| BTA | Position | P-value | Allele | Frequency | Effect | Annotation | Gene |
| --- | --- | --- | --- | --- | --- | --- | --- |
| 1 | 16076434 | 2.03X10^-8^ | G | 0.971 | 0.062 | intergenic |  |
| 1 | 42290007 | 2.20X10^-7^ | A | 0.998 | 0.231 | intergenic |  |
| 1 | 52112166 | 2.04X10^-7^ | A | 0.220 | 0.023 | intergenic |  |
| 2 | 127223857 | 4.57X10^-7^ | C | 0.998 | 0.214 | intergenic |  |
| 2 | 127224628 | 4.57X10^-7^ | A | 0.998 | 0.214 | intergenic |  |
| 2 | 127225475 | 4.57X10^-7^ | A | 0.998 | 0.214 | intergenic |  |
| 2 | 127225505 | 4.57X10^-7^ | A | 0.998 | 0.214 | intergenic |  |
| 2 | 127225827 | 4.57X10^-7^ | C | 0.998 | 0.214 | intergenic |  |
| 2 | 127225838 | 4.57X10^-7^ | A | 0.998 | 0.214 | intergenic |  |
| 2 | 127225918 | 4.57X10^-7^ | T | 0.998 | 0.214 | intergenic |  |
| 2 | 127226191 | 4.57X10^-7^ | G | 0.998 | 0.214 | intergenic |  |
| 2 | 127226621 | 4.57X10^-7^ | T | 0.998 | 0.214 | intergenic |  |
| 2 | 127228011 | 4.57X10^-7^ | G | 0.998 | 0.214 | intergenic |  |
| 2 | 127228241 | 4.57X10^-7^ | A | 0.998 | 0.214 | intergenic |  |
| 2 | 127228691 | 4.57X10^-7^ | A | 0.998 | 0.214 | intergenic |  |
| 2 | 127229803 | 4.57X10^-7^ | A | 0.998 | 0.214 | intergenic |  |
| 2 | 127230765 | 4.57X10^-7^ | C | 0.998 | 0.214 | intergenic |  |
| 2 | 127231566 | 4.57X10^-7^ | C | 0.998 | 0.214 | intergenic |  |
| 2 | 127233893 | 4.57X10^-7^ | C | 0.998 | 0.214 | intergenic |  |
| 2 | 127235771 | 4.57X10^-7^ | A | 0.998 | 0.214 | intergenic |  |
| 2 | 127236142 | 4.57X10^-7^ | G | 0.998 | 0.214 | intergenic |  |
| 2 | 127236257 | 4.57X10^-7^ | A | 0.998 | 0.214 | intergenic |  |
| 2 | 127236544 | 4.57X10^-7^ | G | 0.998 | 0.214 | intergenic |  |
| 2 | 127237510 | 4.57X10^-7^ | G | 0.998 | 0.214 | intergenic |  |
| 2 | 127237571 | 4.57X10^-7^ | T | 0.998 | 0.214 | intergenic |  |
| 2 | 127241840 | 4.57X10^-7^ | G | 0.998 | 0.214 | intergenic |  |
| 2 | 127243352 | 4.57X10^-7^ | C | 0.998 | 0.214 | intergenic |  |
| 2 | 127243670 | 4.57X10^-7^ | G | 0.998 | 0.214 | intergenic |  |
| 2 | 127244366 | 4.57X10^-7^ | T | 0.998 | 0.214 | intergenic |  |
| 2 | 127245377 | 4.57X10^-7^ | G | 0.998 | 0.214 | intergenic |  |
| 2 | 127247840 | 4.57X10^-7^ | C | 0.998 | 0.214 | downstream gene | ENSBTAG00000038104 |
| 2 | 127248819 | 4.57X10^-7^ | T | 0.998 | 0.214 | downstream gene | ENSBTAG00000038104 |
| 2 | 127248858 | 4.57X10^-7^ | T | 0.998 | 0.214 | downstream gene | ENSBTAG00000038104 |
| 2 | 127249241 | 4.57X10^-7^ | C | 0.998 | 0.214 | downstream gene | ENSBTAG00000038104 |
| 2 | 127250269 | 4.57X10^-7^ | C | 0.998 | 0.214 | downstream gene | ENSBTAG00000038104 |
| 2 | 127252636 | 4.57X10^-7^ | C | 0.998 | 0.214 | splice variant | ENSBTAG00000038104 |
| 2 | 127253226 | 1.26X10^-7^ | T | 0.998 | 0.216 | intron | ENSBTAG00000038104 |
| 2 | 127253903 | 1.26X10^-7^ | G | 0.998 | 0.216 | intron | ENSBTAG00000038104 |
| 3 | 20800231 | 9.18X10^-8^ | A | 0.998 | 0.214 | downstream gene | ENSBTAG00000038604 |
| 3 | 58176262 | 1.06X10^-8^ | C | 0.997 | 0.219 | intergenic |  |
| 3 | 63684962 | 9.92X10^-7^ | C | 0.997 | 0.166 | intergenic |  |
| 5 | 10684331 | 1.79X10^-7^ | G | 0.998 | 0.185 | intergenic |  |
| 5 | 14072592 | 2.38E-11 | G | 0.998 | 0.259 | intergenic |  |
| 5 | 14073008 | 1.08X10^-8^ | C | 0.997 | 0.170 | intergenic |  |
| 5 | 70739873 | 2.27X10^-7^ | T | 0.997 | 0.179 | intergenic |  |
| 5 | 70762814 | 4.15X10^-7^ | C | 0.997 | 0.206 | intergenic |  |
| 5 | 110669424 | 6.33X10^-7^ | C | 0.996 | 0.148 | intron | KDELR3 |
| 7 | 30657056 | 3.60X10^-7^ | A | 0.004 | 0.149 | intergenic |  |
| 8 | 8939765 | 6.86X10^-7^ | G | 0.969 | 0.053 | intron | MSRA |
| 9 | 48904868 | 6.15X10^-9^ | T | 0.998 | 0.248 | intron | GRIK2 |
| 11 | 36157469 | 3.94X10^-7^ | A | 0.997 | 0.171 | intergenic |  |
| 11 | 66077015 | 1.88X10^-7^ | G | 0.994 | 0.126 | intergenic |  |
| 11 | 66124814 | 3.00X10^-8^ | C | 0.995 | 0.142 | intergenic |  |
| 11 | 66189393 | 1.03X10^-7^ | G | 0.995 | 0.139 | intergenic |  |
| 11 | 66248110 | 1.03X10^-7^ | A | 0.995 | 0.141 | intergenic |  |
| 11 | 75076003 | 3.28X10^-7^ | G | 0.998 | 0.216 | intron | WDCP |
| 11 | 75865330 | 5.93X10^-7^ | G | 0.026 | 0.052 | intergenic |  |
| 12 | 3992476 | 8.37X10^-7^ | A | 0.952 | 0.046 | intergenic |  |
| 12 | 4019799 | 2.44X10^-7^ | G | 0.954 | 0.048 | intergenic |  |
| 12 | 33805903 | 7.55X10^-7^ | A | 0.996 | 0.131 | intron | ENSBTAG00000019529 |
| 12 | 39991011 | 5.97X10^-7^ | G | 0.993 | 0.108 | intergenic |  |
| 12 | 40571639 | 9.04X10^-7^ | C | 0.996 | 0.137 | intergenic |  |
| 12 | 70173808 | 3.10X10^-7^ | G | 0.018 | 0.072 | upstream gene | ABCC4 |
| 12 | 70186511 | 9.27X10^-7^ | C | 0.995 | 0.129 | intergenic |  |
| 12 | 70186819 | 9.27X10^-7^ | C | 0.995 | 0.129 | intergenic |  |
| 12 | 70186865 | 9.27X10^-7^ | T | 0.995 | 0.129 | intergenic |  |
| 12 | 70186893 | 9.27X10^-7^ | T | 0.995 | 0.129 | intergenic |  |
| 12 | 70187733 | 9.27X10^-7^ | T | 0.995 | 0.129 | intergenic |  |
| 12 | 70187900 | 9.27X10^-7^ | G | 0.995 | 0.129 | intergenic |  |
| 15 | 72327693 | 4.27X10^-7^ | C | 0.998 | 0.215 | intergenic |  |
| 16 | 61284851 | 1.97X10^-7^ | T | 0.997 | 0.164 | intron | RASAL2 |
| 16 | 61285588 | 1.97X10^-7^ | C | 0.997 | 0.164 | intron | RASAL2 |
| 16 | 61285657 | 1.97X10^-7^ | C | 0.997 | 0.164 | intron | RASAL2 |
| 16 | 61337899 | 1.97X10^-7^ | G | 0.997 | 0.164 | intergenic |  |
| 16 | 61355677 | 1.97X10^-7^ | T | 0.997 | 0.164 | intron | TEX35 |
| 16 | 61403299 | 6.46X10^-8^ | G | 0.997 | 0.167 | intergenic |  |
| 16 | 61409522 | 6.46X10^-8^ | G | 0.997 | 0.167 | intergenic |  |
| 16 | 61441788 | 1.41X10^-7^ | C | 0.996 | 0.154 | intergenic |  |
| 16 | 61444038 | 1.41X10^-7^ | G | 0.996 | 0.154 | intergenic |  |
| 16 | 61473116 | 1.41X10^-7^ | C | 0.996 | 0.154 | intergenic |  |
| 16 | 67412575 | 1.19X10^-7^ | A | 0.921 | 0.036 | intron | FAM129A |
| 16 | 67412581 | 9.78X10^-10^ | T | 0.940 | 0.046 | intron | FAM129A |
| 16 | 67413755 | 7.32X10^-8^ | T | 0.939 | 0.040 | intron | FAM129A |
| 17 | 16828764 | 2.04X10^-7^ | C | 0.990 | 0.090 | intergenic |  |
| 17 | 16898348 | 1.91X10^-8^ | T | 0.969 | 0.054 | intergenic |  |
| 17 | 16900106 | 4.78X10^-7^ | G | 0.990 | 0.087 | intergenic |  |
| 17 | 16902192 | 2.97X10^-8^ | A | 0.968 | 0.053 | intergenic |  |
| 17 | 16907959 | 2.97X10^-8^ | T | 0.968 | 0.053 | intergenic |  |
| 17 | 16984691 | 4.78X10^-7^ | T | 0.990 | 0.087 | intergenic |  |
| 17 | 17595339 | 2.62X10^-7^ | A | 0.991 | 0.092 | intron | CLGN |
| 17 | 29867937 | 7.37X10^-7^ | G | 0.974 | 0.055 | upstream gene | PGRMC2 |
| 17 | 29870359 | 7.37X10^-7^ | G | 0.974 | 0.055 | upstream gene | PGRMC2 |
| 18 | 14741066 | 1.13X10^-7^ | G | 0.997 | 0.172 | upstream gene | TCF25 |
| 18 | 14744638 | 1.13X10^-7^ | G | 0.997 | 0.172 | intron | TCF25 |
| 18 | 14747566 | 1.13X10^-7^ | C | 0.997 | 0.172 | missense | TCF25 |
| 18 | 14752240 | 1.13X10^-7^ | C | 0.997 | 0.172 | intron | TCF25 |
| 18 | 14752447 | 3.39X10^-7^ | C | 0.996 | 0.161 | intron | TCF25 |
| 18 | 14753120 | 1.13X10^-7^ | G | 0.997 | 0.172 | intron | TCF25 |
| 18 | 14757740 | 1.13X10^-7^ | C | 0.997 | 0.172 | upstream gene | TUBB3 |
| 19 | 4138272 | 6.06X10^-9^ | C | 0.940 | 0.045 | intergenic |  |
| 19 | 4141067 | 1.41X10^-9^ | C | 0.943 | 0.048 | intergenic |  |
| 19 | 4149672 | 1.59X10^-7^ | C | 0.931 | 0.039 | intergenic |  |
| 19 | 4151309 | 1.63X10^-7^ | G | 0.931 | 0.038 | intergenic |  |
| 19 | 4151794 | 2.00X10^-8^ | T | 0.956 | 0.051 | intergenic |  |
| 19 | 4153524 | 1.68X10^-7^ | C | 0.931 | 0.038 | intergenic |  |
| 19 | 4154165 | 2.55X10^-8^ | G | 0.956 | 0.050 | intergenic |  |
| 19 | 4154626 | 2.55X10^-8^ | G | 0.956 | 0.050 | intergenic |  |
| 19 | 5973158 | 6.83X10^-7^ | A | 0.998 | 0.209 | intergenic |  |
| 19 | 14389393 | 3.84X10^-7^ | C | 0.002 | 0.189 | intergenic |  |
| 19 | 14396120 | 3.84X10^-7^ | G | 0.002 | 0.189 | intergenic |  |
| 20 | 62522235 | 7.32X10^-7^ | G | 0.993 | 0.107 | intergenic |  |
| 23 | 14833068 | 7.34X10^-7^ | C | 0.997 | 0.196 | intergenic |  |
| 23 | 19627820 | 5.24X10^-7^ | C | 0.996 | 0.164 | intron | RCAN2 |
| 23 | 19653076 | 1.90X10^-7^ | T | 0.997 | 0.174 | intron | RCAN2 |
| 23 | 19671383 | 1.90X10^-7^ | C | 0.997 | 0.174 | upstream gene | RCAN2 |
| 24 | 6616896 | 8.04X10^-7^ | A | 0.982 | 0.071 | intergenic |  |
| 25 | 5098855 | 5.05X10^-7^ | G | 0.998 | 0.185 | intergenic |  |
| 25 | 15936181 | 9.37X10^-7^ | T | 0.948 | 0.041 | intron | XYLT1 |
| 27 | 16166731 | 2.63X10^-10^ | T | 0.997 | 0.206 | intergenic |  |
| 27 | 23474058 | 7.94X10^-7^ | G | 0.913 | 0.034 | intron | ENSBTAG00000000357 |
