# Supplementary material for: Variance components for bovine tuberculosis infection and multi-breed genome-wide association analysis using imputed whole genome sequence data
Source: PLoS One. 2019 Feb 14;14(2):e0212067. doi: 10.1371/journal.pone.0212067 (PMC6375599; doi:10.1371/journal.pone.0212067)
Supplement: S5 Table — (DOCX) [file pone.0212067.s006.docx]

**Table S5.** Chromosome (BTA), position, P-value, the favorable allele, the frequency of the favorable allele, substitution effect of the favorable allele, annotation, and gene for the 325 single nucleotide polymorphisms associated with bovine tuberculosis infection in the within-breed analysis of Charolais bulls (P < 1 x 10^-6^)

| BTA | Position | P-value | Allele | Frequency | Effect | Annotation | Gene |
| --- | --- | --- | --- | --- | --- | --- | --- |
| 1 | 85947826 | 1.96X10^-7^ | C | 0.998 | 0.186 | upstream gene | ENSBTAG00000043803 |
| 1 | 85950766 | 1.96X10^-7^ | G | 0.998 | 0.186 | upstream gene | ENSBTAG00000043803 |
| 1 | 85978716 | 1.96X10^-7^ | G | 0.998 | 0.186 | intergenic |  |
| 1 | 95426117 | 8.17X10^-7^ | G | 0.996 | 0.125 | intergenic |  |
| 1 | 95448122 | 2.24X10^-7^ | A | 0.985 | 0.076 | intergenic |  |
| 3 | 48518616 | 1.16X10^-7^ | T | 0.997 | 0.136 | intergenic |  |
| 4 | 1711208 | 4.43X10^-7^ | G | 0.996 | 0.15 | intergenic |  |
| 4 | 1745977 | 8.64X10^-8^ | C | 0.997 | 0.164 | intergenic |  |
| 4 | 1772030 | 6.07E-10 | T | 0.998 | 0.221 | intergenic |  |
| 4 | 1826499 | 1.03X10^-8^ | G | 0.998 | 0.196 | intergenic |  |
| 4 | 1829115 | 1.03X10^-8^ | G | 0.998 | 0.196 | intergenic |  |
| 4 | 1838794 | 1.39X10^-9^ | C | 0.998 | 0.216 | intergenic |  |
| 4 | 1883110 | 9.90X10^-7^ | C | 0.996 | 0.141 | intergenic |  |
| 4 | 1987658 | 3.77X10^-7^ | C | 0.996 | 0.15 | intergenic |  |
| 4 | 2017985 | 2.05X10^-8^ | T | 0.997 | 0.176 | intergenic |  |
| 4 | 7204214 | 6.91X10^-7^ | A | 0.993 | 0.095 | intron | ABCA13 |
| 4 | 7204230 | 6.91X10^-7^ | T | 0.993 | 0.095 | intron | ABCA13 |
| 4 | 72806317 | 2.81X10^-7^ | A | 0.996 | 0.123 | intergenic |  |
| 5 | 104575856 | 1.31X10^-8^ | G | 0.935 | 0.041 | intergenic |  |
| 5 | 104578251 | 2.01X10^-8^ | T | 0.937 | 0.041 | intergenic |  |
| 5 | 104580658 | 2.69X10^-8^ | A | 0.938 | 0.041 | intergenic |  |
| 5 | 104587778 | 6.34X10^-7^ | C | 0.94 | 0.037 | intergenic |  |
| 5 | 104593479 | 3.21X10^-7^ | A | 0.939 | 0.038 | intergenic |  |
| 5 | 111010187 | 1.54X10^-7^ | A | 0.998 | 0.182 | intergenic |  |
| 6 | 14931047 | 9.60X10^-7^ | C | 0.998 | 0.153 | intergenic |  |
| 6 | 14953334 | 1.33X10^-7^ | C | 0.998 | 0.182 | intergenic |  |
| 6 | 70808959 | 5.90X10^-9^ | A | 0.998 | 0.232 | intergenic |  |
| 6 | 113985228 | 2.80X10^-7^ | C | 0.998 | 0.177 | intergenic |  |
| 6 | 114006331 | 2.80X10^-7^ | T | 0.998 | 0.177 | intergenic |  |
| 6 | 114006335 | 2.80X10^-7^ | T | 0.998 | 0.177 | intergenic |  |
| 6 | 114006358 | 2.80X10^-7^ | T | 0.998 | 0.177 | intergenic |  |
| 7 | 40751593 | 1.92X10^-7^ | A | 0.997 | 0.155 | intergenic |  |
| 7 | 73255240 | 3.23X10^-7^ | A | 0.081 | 0.032 | intergenic |  |
| 7 | 73255247 | 4.51X10^-7^ | A | 0.081 | 0.031 | intergenic |  |
| 8 | 4405061 | 1.69X10^-7^ | T | 0.003 | 0.158 | intron | GALNTL6 |
| 8 | 4405688 | 1.69X10^-7^ | C | 0.003 | 0.158 | intron | GALNTL6 |
| 8 | 36561299 | 2.93X10^-9^ | G | 0.997 | 0.18 | intergenic |  |
| 8 | 105232215 | 3.97X10^-7^ | G | 0.998 | 0.198 | intron | AKNA |
| 9 | 15405248 | 7.37X10^-7^ | C | 0.996 | 0.14 | intergenic |  |
| 9 | 88918083 | 8.79X10^-7^ | G | 0.98 | 0.061 | intergenic |  |
| 10 | 78630522 | 6.18X10^-7^ | C | 0.983 | 0.062 | intergenic |  |
| 10 | 78631825 | 4.80X10^-7^ | G | 0.984 | 0.063 | intergenic |  |
| 10 | 78634440 | 4.80X10^-7^ | T | 0.984 | 0.063 | intergenic |  |
| 10 | 78635957 | 3.93X10^-7^ | G | 0.98 | 0.058 | intergenic |  |
| 10 | 78635979 | 3.93X10^-7^ | T | 0.98 | 0.058 | intergenic |  |
| 10 | 78636684 | 4.55X10^-7^ | T | 0.982 | 0.062 | intergenic |  |
| 10 | 78639251 | 1.10X10^-7^ | C | 0.98 | 0.062 | intergenic |  |
| 10 | 78639937 | 1.10X10^-7^ | C | 0.98 | 0.062 | intergenic |  |
| 10 | 78640279 | 5.31X10^-8^ | A | 0.983 | 0.067 | intergenic |  |
| 10 | 78642794 | 5.31X10^-8^ | G | 0.983 | 0.067 | intergenic |  |
| 10 | 78646435 | 1.10X10^-7^ | C | 0.98 | 0.062 | intergenic |  |
| 10 | 78647711 | 3.79X10^-8^ | C | 0.982 | 0.068 | intergenic |  |
| 10 | 78650177 | 3.79X10^-8^ | A | 0.982 | 0.068 | intergenic |  |
| 10 | 78655994 | 3.79X10^-8^ | T | 0.982 | 0.068 | intergenic |  |
| 10 | 78657618 | 1.34X10^-8^ | T | 0.985 | 0.075 | intergenic |  |
| 10 | 78658008 | 1.52X10^-7^ | T | 0.985 | 0.07 | intergenic |  |
| 10 | 78658197 | 3.79X10^-8^ | T | 0.982 | 0.068 | intergenic |  |
| 10 | 78658234 | 1.34X10^-8^ | C | 0.985 | 0.075 | intergenic |  |
| 10 | 78658241 | 3.79X10^-8^ | T | 0.982 | 0.068 | intergenic |  |
| 10 | 78658664 | 3.79X10^-8^ | A | 0.982 | 0.068 | intergenic |  |
| 10 | 78658713 | 3.79X10^-8^ | G | 0.982 | 0.068 | intergenic |  |
| 10 | 78658738 | 4.12X10^-8^ | A | 0.981 | 0.068 | intergenic |  |
| 10 | 78658899 | 3.79X10^-8^ | C | 0.982 | 0.068 | intergenic |  |
| 10 | 78659356 | 3.79X10^-8^ | T | 0.982 | 0.068 | intergenic |  |
| 10 | 78659430 | 3.79X10^-8^ | G | 0.982 | 0.068 | intergenic |  |
| 10 | 78659600 | 3.79X10^-8^ | T | 0.982 | 0.068 | intergenic |  |
| 10 | 78659623 | 3.79X10^-8^ | C | 0.982 | 0.068 | intergenic |  |
| 10 | 78659711 | 3.79X10^-8^ | A | 0.982 | 0.068 | intergenic |  |
| 10 | 78660967 | 3.79X10^-8^ | T | 0.982 | 0.068 | intergenic |  |
| 11 | 103912851 | 2.09X10^-7^ | T | 0.984 | 0.07 | upstream gene | PMPCA |
| 11 | 103917027 | 3.41X10^-7^ | G | 0.985 | 0.071 | intron | PMPCA |
| 11 | 103918361 | 3.41X10^-7^ | G | 0.985 | 0.071 | intron | PMPCA |
| 11 | 103963912 | 4.07X10^-7^ | C | 0.986 | 0.072 | synonymous | SEC16A |
| 12 | 14242742 | 8.47X10^-7^ | C | 0.956 | 0.043 | intergenic |  |
| 12 | 20037737 | 5.94X10^-8^ | T | 0.997 | 0.174 | intergenic |  |
| 12 | 20049260 | 5.44X10^-8^ | A | 0.996 | 0.17 | intergenic |  |
| 12 | 20083605 | 1.13X10^-7^ | A | 0.996 | 0.163 | intergenic |  |
| 12 | 20090015 | 1.13X10^-7^ | G | 0.996 | 0.163 | intergenic |  |
| 12 | 20139412 | 9.05X10^-7^ | T | 0.997 | 0.161 | intergenic |  |
| 12 | 20139419 | 9.05X10^-7^ | T | 0.997 | 0.161 | intergenic |  |
| 13 | 14439510 | 9.83X10^-7^ | T | 0.998 | 0.212 | intergenic |  |
| 13 | 20944126 | 5.17X10^-8^ | G | 0.998 | 0.204 | intron | ENSBTAG00000037969 |
| 14 | 41001133 | 1.43X10^-8^ | A | 0.998 | 0.199 | intergenic |  |
| 14 | 41001943 | 1.43X10^-8^ | C | 0.998 | 0.199 | intergenic |  |
| 14 | 41007125 | 1.40X10^-7^ | C | 0.998 | 0.18 | intergenic |  |
| 14 | 63880770 | 9.47X10^-7^ | G | 0.993 | 0.097 | intergenic |  |
| 14 | 63884449 | 9.47X10^-7^ | G | 0.993 | 0.097 | intergenic |  |
| 15 | 1894388 | 5.38X10^-7^ | C | 0.998 | 0.192 | downstream gene | KBTBD3 |
| 15 | 24095390 | 9.00X10^-7^ | T | 0.997 | 0.152 | intron | NCAM1 |
| 15 | 26875567 | 2.64X10^-7^ | A | 0.998 | 0.178 | intergenic |  |
| 15 | 26875580 | 8.28X10^-7^ | T | 0.996 | 0.127 | intergenic |  |
| 15 | 37486166 | 4.88X10^-7^ | C | 0.987 | 0.073 | intergenic |  |
| 15 | 73765014 | 2.28X10^-7^ | G | 0.977 | 0.06 | intergenic |  |
| 16 | 79608846 | 8.30X10^-7^ | T | 0.997 | 0.166 | intergenic |  |
| 16 | 79609614 | 8.30X10^-7^ | C | 0.997 | 0.166 | intergenic |  |
| 16 | 79630527 | 3.36X10^-8^ | G | 0.998 | 0.207 | intergenic |  |
| 16 | 79630988 | 3.36X10^-8^ | G | 0.998 | 0.207 | intergenic |  |
| 16 | 79632638 | 3.36X10^-8^ | A | 0.998 | 0.207 | intergenic |  |
| 16 | 79633043 | 3.36X10^-8^ | A | 0.998 | 0.207 | intergenic |  |
| 16 | 79633064 | 3.36X10^-8^ | A | 0.998 | 0.207 | intergenic |  |
| 16 | 79633149 | 3.36X10^-8^ | T | 0.998 | 0.207 | intergenic |  |
| 16 | 79633281 | 3.36X10^-8^ | A | 0.998 | 0.207 | intergenic |  |
| 16 | 79633419 | 3.36X10^-8^ | C | 0.998 | 0.207 | intergenic |  |
| 16 | 79633533 | 3.36X10^-8^ | G | 0.998 | 0.207 | intergenic |  |
| 16 | 79633540 | 4.50X10^-9^ | A | 0.998 | 0.213 | intergenic |  |
| 16 | 79633566 | 3.36X10^-8^ | A | 0.998 | 0.207 | intergenic |  |
| 16 | 79633580 | 3.36X10^-8^ | T | 0.998 | 0.207 | intergenic |  |
| 16 | 79633593 | 3.36X10^-8^ | G | 0.998 | 0.207 | intergenic |  |
| 16 | 79633678 | 3.36X10^-8^ | C | 0.998 | 0.207 | intergenic |  |
| 16 | 79633733 | 3.36X10^-8^ | T | 0.998 | 0.207 | intergenic |  |
| 16 | 79633896 | 3.36X10^-8^ | C | 0.998 | 0.207 | intergenic |  |
| 16 | 79633963 | 3.36X10^-8^ | A | 0.998 | 0.207 | intergenic |  |
| 16 | 79633992 | 3.36X10^-8^ | T | 0.998 | 0.207 | intergenic |  |
| 16 | 79633998 | 3.36X10^-8^ | G | 0.998 | 0.207 | intergenic |  |
| 16 | 79634009 | 3.36X10^-8^ | T | 0.998 | 0.207 | intergenic |  |
| 16 | 79634066 | 3.36X10^-8^ | T | 0.998 | 0.207 | intergenic |  |
| 16 | 79634131 | 3.36X10^-8^ | A | 0.998 | 0.207 | intergenic |  |
| 16 | 79634340 | 3.36X10^-8^ | T | 0.998 | 0.207 | intergenic |  |
| 16 | 79634440 | 3.36X10^-8^ | G | 0.998 | 0.207 | intergenic |  |
| 16 | 79634533 | 3.36X10^-8^ | C | 0.998 | 0.207 | intergenic |  |
| 16 | 79634729 | 3.36X10^-8^ | C | 0.998 | 0.207 | intergenic |  |
| 16 | 79634931 | 8.67X10^-7^ | A | 0.997 | 0.141 | intergenic |  |
| 16 | 79635030 | 3.36X10^-8^ | C | 0.998 | 0.207 | intergenic |  |
| 16 | 79635186 | 3.36X10^-8^ | G | 0.998 | 0.207 | intergenic |  |
| 16 | 79635242 | 3.36X10^-8^ | A | 0.998 | 0.207 | intergenic |  |
| 16 | 79635411 | 3.36X10^-8^ | A | 0.998 | 0.207 | intergenic |  |
| 16 | 79635641 | 3.36X10^-8^ | T | 0.998 | 0.207 | intergenic |  |
| 16 | 79635812 | 3.36X10^-8^ | C | 0.998 | 0.207 | intergenic |  |
| 16 | 79636133 | 3.36X10^-8^ | A | 0.998 | 0.207 | intergenic |  |
| 16 | 79636196 | 3.36X10^-8^ | G | 0.998 | 0.207 | intergenic |  |
| 16 | 79636353 | 3.36X10^-8^ | T | 0.998 | 0.207 | intergenic |  |
| 16 | 79636577 | 3.36X10^-8^ | T | 0.998 | 0.207 | intergenic |  |
| 16 | 79636802 | 3.36X10^-8^ | C | 0.998 | 0.207 | intergenic |  |
| 16 | 79636950 | 3.36X10^-8^ | A | 0.998 | 0.207 | intergenic |  |
| 16 | 79637074 | 3.36X10^-8^ | C | 0.998 | 0.207 | intergenic |  |
| 16 | 79637670 | 3.36X10^-8^ | G | 0.998 | 0.207 | intergenic |  |
| 16 | 79637788 | 3.36X10^-8^ | T | 0.998 | 0.207 | intergenic |  |
| 16 | 79637800 | 3.36X10^-8^ | T | 0.998 | 0.207 | intergenic |  |
| 16 | 79637963 | 3.36X10^-8^ | T | 0.998 | 0.207 | intergenic |  |
| 16 | 79637990 | 3.36X10^-8^ | G | 0.998 | 0.207 | intergenic |  |
| 16 | 79638200 | 3.36X10^-8^ | C | 0.998 | 0.207 | intergenic |  |
| 16 | 79638246 | 3.36X10^-8^ | C | 0.998 | 0.207 | intergenic |  |
| 16 | 79638706 | 3.36X10^-8^ | A | 0.998 | 0.207 | intergenic |  |
| 16 | 79639519 | 3.36X10^-8^ | G | 0.998 | 0.207 | intergenic |  |
| 16 | 79639799 | 8.67X10^-7^ | T | 0.997 | 0.141 | intergenic |  |
| 16 | 79640364 | 3.36X10^-8^ | T | 0.998 | 0.207 | intergenic |  |
| 16 | 79641230 | 3.36X10^-8^ | G | 0.998 | 0.207 | intergenic |  |
| 16 | 79641303 | 3.36X10^-8^ | C | 0.998 | 0.207 | intergenic |  |
| 16 | 79642940 | 3.36X10^-8^ | C | 0.998 | 0.207 | intergenic |  |
| 16 | 79642953 | 3.36X10^-8^ | T | 0.998 | 0.207 | intergenic |  |
| 16 | 79643153 | 3.36X10^-8^ | G | 0.998 | 0.207 | intergenic |  |
| 16 | 79643388 | 3.36X10^-8^ | A | 0.998 | 0.207 | intergenic |  |
| 16 | 79643580 | 3.36X10^-8^ | A | 0.998 | 0.207 | intergenic |  |
| 16 | 79643686 | 3.36X10^-8^ | G | 0.998 | 0.207 | intergenic |  |
| 16 | 79643713 | 3.36X10^-8^ | A | 0.998 | 0.207 | intergenic |  |
| 16 | 79643758 | 3.36X10^-8^ | T | 0.998 | 0.207 | intergenic |  |
| 16 | 79643762 | 3.36X10^-8^ | A | 0.998 | 0.207 | intergenic |  |
| 16 | 79643882 | 3.36X10^-8^ | C | 0.998 | 0.207 | intergenic |  |
| 16 | 79643957 | 3.36X10^-8^ | G | 0.998 | 0.207 | intergenic |  |
| 16 | 79644018 | 3.36X10^-8^ | C | 0.998 | 0.207 | intergenic |  |
| 16 | 79644079 | 3.36X10^-8^ | A | 0.998 | 0.207 | intergenic |  |
| 16 | 79644186 | 3.36X10^-8^ | A | 0.998 | 0.207 | intergenic |  |
| 16 | 79644266 | 3.36X10^-8^ | G | 0.998 | 0.207 | intergenic |  |
| 16 | 79646374 | 4.71X10^-7^ | A | 0.996 | 0.137 | intergenic |  |
| 16 | 79646398 | 8.67X10^-7^ | A | 0.997 | 0.141 | intergenic |  |
| 16 | 79646481 | 8.67X10^-7^ | C | 0.997 | 0.141 | intergenic |  |
| 16 | 79646518 | 4.71X10^-7^ | T | 0.996 | 0.137 | intergenic |  |
| 16 | 79646733 | 8.67X10^-7^ | T | 0.997 | 0.141 | intergenic |  |
| 16 | 79646796 | 8.67X10^-7^ | G | 0.997 | 0.141 | intergenic |  |
| 16 | 79646869 | 4.71X10^-7^ | A | 0.996 | 0.137 | intergenic |  |
| 16 | 79646874 | 3.36X10^-8^ | C | 0.998 | 0.207 | intergenic |  |
| 16 | 79646928 | 4.71X10^-7^ | A | 0.996 | 0.137 | intergenic |  |
| 16 | 79647037 | 4.71X10^-7^ | A | 0.996 | 0.137 | intergenic |  |
| 16 | 79647241 | 4.71X10^-7^ | A | 0.996 | 0.137 | intergenic |  |
| 16 | 79647428 | 8.67X10^-7^ | A | 0.997 | 0.141 | intergenic |  |
| 16 | 79647738 | 3.36X10^-8^ | C | 0.998 | 0.207 | intergenic |  |
| 16 | 79647778 | 3.25X10^-8^ | G | 0.997 | 0.191 | intergenic |  |
| 16 | 79647968 | 3.25X10^-8^ | G | 0.997 | 0.191 | intergenic |  |
| 16 | 79648004 | 3.25X10^-8^ | G | 0.997 | 0.191 | intergenic |  |
| 16 | 79648240 | 3.36X10^-8^ | T | 0.998 | 0.207 | intergenic |  |
| 16 | 79648462 | 3.25X10^-8^ | T | 0.997 | 0.191 | intergenic |  |
| 16 | 79648492 | 3.25X10^-8^ | A | 0.997 | 0.191 | intergenic |  |
| 16 | 79648705 | 3.25X10^-8^ | G | 0.997 | 0.191 | intergenic |  |
| 16 | 79648715 | 3.36X10^-8^ | A | 0.998 | 0.207 | intergenic |  |
| 16 | 79648719 | 3.36X10^-8^ | G | 0.998 | 0.207 | intergenic |  |
| 16 | 79648723 | 3.25X10^-8^ | A | 0.997 | 0.191 | intergenic |  |
| 16 | 79648974 | 3.36X10^-8^ | G | 0.998 | 0.207 | intergenic |  |
| 16 | 79649443 | 3.36X10^-8^ | G | 0.998 | 0.207 | intergenic |  |
| 16 | 79649457 | 3.25X10^-8^ | A | 0.997 | 0.191 | intergenic |  |
| 16 | 79649461 | 3.25X10^-8^ | A | 0.997 | 0.191 | intergenic |  |
| 16 | 79649464 | 3.25X10^-8^ | T | 0.997 | 0.191 | intergenic |  |
| 16 | 79649476 | 3.25X10^-8^ | A | 0.997 | 0.191 | intergenic |  |
| 16 | 79649540 | 3.36X10^-8^ | G | 0.998 | 0.207 | intergenic |  |
| 16 | 79649565 | 3.25X10^-8^ | T | 0.997 | 0.191 | intergenic |  |
| 16 | 79649590 | 3.25X10^-8^ | G | 0.997 | 0.191 | intergenic |  |
| 16 | 79649692 | 3.25X10^-8^ | T | 0.997 | 0.191 | intergenic |  |
| 16 | 79649733 | 3.25X10^-8^ | T | 0.997 | 0.191 | intergenic |  |
| 16 | 79649743 | 3.25X10^-8^ | T | 0.997 | 0.191 | intergenic |  |
| 16 | 79649752 | 3.25X10^-8^ | A | 0.997 | 0.191 | intergenic |  |
| 16 | 79649835 | 3.25X10^-8^ | A | 0.997 | 0.191 | intergenic |  |
| 16 | 79649839 | 3.25X10^-8^ | A | 0.997 | 0.191 | intergenic |  |
| 16 | 79649912 | 3.25X10^-8^ | A | 0.997 | 0.191 | intergenic |  |
| 16 | 79650185 | 3.25X10^-8^ | C | 0.997 | 0.191 | intergenic |  |
| 16 | 79650274 | 3.25X10^-8^ | C | 0.997 | 0.191 | intergenic |  |
| 16 | 79650405 | 3.25X10^-8^ | A | 0.997 | 0.191 | intergenic |  |
| 16 | 79650529 | 3.25X10^-8^ | G | 0.997 | 0.191 | intergenic |  |
| 16 | 79650877 | 3.25X10^-8^ | A | 0.997 | 0.191 | intergenic |  |
| 16 | 79651218 | 3.36X10^-8^ | T | 0.998 | 0.207 | intergenic |  |
| 16 | 79651287 | 3.25X10^-8^ | G | 0.997 | 0.191 | intergenic |  |
| 16 | 79651362 | 3.25X10^-8^ | C | 0.997 | 0.191 | intergenic |  |
| 16 | 79651561 | 3.25X10^-8^ | C | 0.997 | 0.191 | intergenic |  |
| 16 | 79652906 | 2.32X10^-7^ | T | 0.997 | 0.164 | intergenic |  |
| 16 | 79653560 | 8.07X10^-8^ | T | 0.996 | 0.159 | intergenic |  |
| 17 | 53606381 | 2.60X10^-7^ | A | 0.994 | 0.114 | intron | NCOR2 |
| 17 | 53648559 | 7.88X10^-7^ | C | 0.994 | 0.111 | intron | NCOR2 |
| 18 | 37928982 | 9.72X10^-7^ | A | 0.994 | 0.11 | intergenic |  |
| 19 | 7097773 | 2.92X10^-8^ | C | 0.98 | 0.07 | intergenic |  |
| 19 | 7114847 | 2.92X10^-8^ | C | 0.98 | 0.07 | intergenic |  |
| 19 | 7128527 | 8.91X10^-9^ | G | 0.984 | 0.082 | intergenic |  |
| 19 | 7130557 | 8.91X10^-9^ | G | 0.984 | 0.082 | intergenic |  |
| 19 | 7130684 | 8.91X10^-9^ | T | 0.984 | 0.082 | intergenic |  |
| 19 | 7132396 | 8.91X10^-9^ | T | 0.984 | 0.082 | intergenic |  |
| 19 | 7135081 | 8.91X10^-9^ | C | 0.984 | 0.082 | intergenic |  |
| 19 | 7138106 | 8.91X10^-9^ | C | 0.984 | 0.082 | intergenic |  |
| 19 | 7140347 | 8.91X10^-9^ | G | 0.984 | 0.082 | intergenic |  |
| 19 | 7141522 | 8.91X10^-9^ | C | 0.984 | 0.082 | intergenic |  |
| 19 | 7147428 | 8.91X10^-9^ | G | 0.984 | 0.082 | intergenic |  |
| 19 | 7149748 | 8.91X10^-9^ | T | 0.984 | 0.082 | intergenic |  |
| 19 | 7152247 | 8.91X10^-9^ | T | 0.984 | 0.082 | intergenic |  |
| 19 | 7152252 | 8.91X10^-9^ | T | 0.984 | 0.082 | intergenic |  |
| 19 | 18979316 | 6.62X10^-7^ | G | 0.998 | 0.164 | intron | NF1 |
| 19 | 19007714 | 8.59X10^-7^ | G | 0.997 | 0.148 | intron | NF1 |
| 19 | 22772044 | 2.17X10^-7^ | G | 0.995 | 0.113 | intron | VPS53 |
| 20 | 56422622 | 7.64X10^-7^ | T | 0.995 | 0.121 | intron | MYO10 |
| 20 | 56431229 | 7.64X10^-7^ | C | 0.995 | 0.121 | synonymous | MYO10 |
| 20 | 56431649 | 7.64X10^-7^ | A | 0.995 | 0.121 | intron | MYO10 |
| 20 | 56434117 | 7.64X10^-7^ | A | 0.995 | 0.121 | intron | MYO10 |
| 20 | 61422564 | 1.12X10^-7^ | A | 0.998 | 0.159 | intergenic |  |
| 20 | 61431545 | 3.30X10^-8^ | G | 0.997 | 0.153 | intergenic |  |
| 20 | 62036334 | 8.62X10^-7^ | T | 0.998 | 0.152 | intron | CTNND2 |
| 20 | 62135121 | 9.91X10^-7^ | A | 0.998 | 0.137 | intron | CTNND2 |
| 20 | 63144429 | 5.09X10^-7^ | G | 0.998 | 0.127 | intergenic |  |
| 21 | 60963049 | 1.78X10^-7^ | T | 0.996 | 0.131 | intron | ENSBTAG00000007043 |
| 21 | 60978987 | 1.78X10^-7^ | G | 0.996 | 0.131 | intron | ENSBTAG00000007043 |
| 21 | 60996900 | 1.78X10^-7^ | G | 0.996 | 0.131 | intron | ENSBTAG00000007041 |
| 21 | 61015254 | 1.78X10^-7^ | C | 0.996 | 0.131 | intergenic |  |
| 22 | 33274015 | 2.36X10^-7^ | T | 0.998 | 0.197 | intron | FAM19A1 |
| 23 | 20321770 | 4.37X10^-7^ | T | 0.997 | 0.158 | intergenic |  |
| 23 | 25011047 | 9.18X10^-7^ | C | 0.175 | 0.022 | intron | ICK |
| 23 | 25011308 | 9.18X10^-7^ | T | 0.175 | 0.022 | intron | ICK |
| 23 | 25011313 | 9.18X10^-7^ | A | 0.175 | 0.022 | intron | ICK |
| 23 | 25011926 | 9.18X10^-7^ | C | 0.175 | 0.022 | intron | ICK |
| 23 | 25013617 | 9.18X10^-7^ | T | 0.175 | 0.022 | intron | ICK |
| 23 | 25013626 | 9.18X10^-7^ | T | 0.175 | 0.022 | intron | ICK |
| 23 | 25014695 | 9.18X10^-7^ | T | 0.175 | 0.022 | intron | ICK |
| 23 | 25041875 | 1.48X10^-7^ | T | 0.2 | 0.022 | intron | FBXO9 |
| 23 | 25042960 | 3.50X10^-7^ | G | 0.214 | 0.021 | intron | FBXO9 |
| 23 | 25043084 | 2.75X10^-7^ | A | 0.209 | 0.021 | intron | FBXO9 |
| 24 | 19061089 | 1.25X10^-7^ | C | 0.995 | 0.137 | intergenic |  |
| 24 | 19076281 | 1.44X10^-8^ | A | 0.996 | 0.176 | intergenic |  |
| 24 | 19080411 | 6.77X10^-10^ | G | 0.997 | 0.199 | intergenic |  |
| 24 | 45112447 | 4.43X10^-7^ | G | 0.961 | 0.044 | intron | SETBP1 |
| 24 | 45127038 | 3.03X10^-7^ | G | 0.957 | 0.043 | intron | SETBP1 |
| 24 | 45136965 | 9.45X10^-7^ | A | 0.966 | 0.045 | intron | SETBP1 |
| 24 | 45137503 | 4.83X10^-7^ | T | 0.966 | 0.046 | intron | SETBP1 |
| 24 | 45138217 | 4.83X10^-7^ | A | 0.966 | 0.046 | intron | SETBP1 |
| 24 | 58060372 | 3.18X10^-7^ | G | 0.998 | 0.175 | intergenic |  |
| 24 | 58060458 | 3.18X10^-7^ | C | 0.998 | 0.175 | intergenic |  |
| 24 | 58078542 | 6.65X10^-7^ | G | 0.997 | 0.153 | intergenic |  |
| 24 | 58079996 | 6.65X10^-7^ | G | 0.997 | 0.153 | intergenic |  |
| 24 | 58081327 | 6.65X10^-7^ | C | 0.997 | 0.153 | intergenic |  |
| 24 | 58082279 | 6.65X10^-7^ | G | 0.997 | 0.153 | intergenic |  |
| 25 | 19296509 | 2.53X10^-7^ | G | 0.997 | 0.164 | intron | CRYM |
| 26 | 11117228 | 1.25X10^-7^ | A | 0.995 | 0.111 | upstream gene | IFIT5 |
| 26 | 26367730 | 1.67X10^-7^ | C | 0.995 | 0.126 | intron | ENSBTAG00000004612 |
| 26 | 26372120 | 1.67X10^-7^ | T | 0.995 | 0.126 | intron | ENSBTAG00000004612 |
| 26 | 26376215 | 2.09X10^-8^ | G | 0.996 | 0.148 | intron | ENSBTAG00000004612 |
| 26 | 26387558 | 1.67X10^-7^ | G | 0.995 | 0.126 | intron | ENSBTAG00000004612 |
| 26 | 26391167 | 1.67X10^-7^ | G | 0.995 | 0.126 | intron | ENSBTAG00000004612 |
| 27 | 2694237 | 2.14X10^-7^ | G | 0.998 | 0.194 | intergenic |  |
| 27 | 2717205 | 2.14X10^-7^ | A | 0.998 | 0.194 | intergenic |  |
| 27 | 2717496 | 2.14X10^-7^ | C | 0.998 | 0.194 | intergenic |  |
| 27 | 2721088 | 2.14X10^-7^ | A | 0.998 | 0.194 | intergenic |  |
| 28 | 1002087 | 8.36X10^-7^ | A | 0.998 | 0.193 | intergenic |  |
| 28 | 1013145 | 8.36X10^-7^ | A | 0.998 | 0.193 | intergenic |  |
| 28 | 1015329 | 8.36X10^-7^ | A | 0.998 | 0.193 | intergenic |  |
| 28 | 1019269 | 8.36X10^-7^ | A | 0.998 | 0.193 | intergenic |  |
| 28 | 1268156 | 2.97X10^-7^ | G | 0.998 | 0.204 | intron | GALNT2 |
| 28 | 20792032 | 1.70X10^-7^ | T | 0.998 | 0.175 | intergenic |  |
| 28 | 24349963 | 1.30X10^-8^ | T | 0.997 | 0.165 | intergenic |  |
| 28 | 30996560 | 6.20X10^-8^ | T | 0.997 | 0.161 | intron | DUPD1 |
| 28 | 30999070 | 6.20X10^-8^ | A | 0.997 | 0.161 | intron | DUPD1 |
| 28 | 31000870 | 6.20X10^-8^ | G | 0.997 | 0.161 | intron | DUPD1 |
| 28 | 31003907 | 6.20X10^-8^ | G | 0.997 | 0.161 | intron | DUPD1 |
| 28 | 31080862 | 4.57X10^-7^ | A | 0.997 | 0.146 | intergenic |  |
| 28 | 31094799 | 4.57X10^-7^ | A | 0.997 | 0.146 | upstream gene | SAMD8 |
| 28 | 36403645 | 2.09X10^-7^ | G | 0.996 | 0.141 | intergenic |  |
| 28 | 37709454 | 6.92X10^-7^ | G | 0.996 | 0.134 | intergenic |  |
| 28 | 37713495 | 6.92X10^-7^ | G | 0.996 | 0.134 | intergenic |  |
| 28 | 37728127 | 6.92X10^-7^ | C | 0.996 | 0.134 | intergenic |  |
| 28 | 37731758 | 1.94X10^-7^ | G | 0.997 | 0.146 | intergenic |  |
| 28 | 37734200 | 3.75X10^-8^ | C | 0.997 | 0.159 | intergenic |  |
| 28 | 37747811 | 1.75X10^-7^ | T | 0.997 | 0.158 | intergenic |  |
| 28 | 38014938 | 1.83X10^-7^ | G | 0.997 | 0.147 | intergenic |  |
| 28 | 38056764 | 1.83X10^-7^ | G | 0.997 | 0.147 | intergenic |  |
| 28 | 38068910 | 3.53X10^-7^ | T | 0.997 | 0.152 | intergenic |  |
| 28 | 38073976 | 3.53X10^-7^ | A | 0.997 | 0.152 | intergenic |  |
| 28 | 38077690 | 6.64X10^-8^ | A | 0.998 | 0.165 | intergenic |  |
| 28 | 38088795 | 3.68X10^-7^ | G | 0.997 | 0.148 | intergenic |  |
| 28 | 38095190 | 2.05X10^-7^ | G | 0.996 | 0.137 | intergenic |  |
| 28 | 38096736 | 3.68X10^-7^ | A | 0.997 | 0.148 | intergenic |  |
| 28 | 39030627 | 3.69X10^-7^ | A | 0.997 | 0.144 | intergenic |  |
| 28 | 39080987 | 3.17X10^-7^ | G | 0.998 | 0.154 | intergenic |  |
| 28 | 39108838 | 3.17X10^-7^ | G | 0.998 | 0.154 | intergenic |  |
| 28 | 39124552 | 3.17X10^-7^ | T | 0.998 | 0.154 | intergenic |  |
| 28 | 39240208 | 4.20X10^-7^ | A | 0.997 | 0.13 | intergenic |  |
| 28 | 39758877 | 1.29X10^-7^ | T | 0.996 | 0.14 | intergenic |  |
| 29 | 9037306 | 2.04X10^-7^ | A | 0.997 | 0.165 | intron | ME3 |
| 29 | 15813829 | 7.61X10^-9^ | G | 0.998 | 0.23 | intergenic |  |
| 29 | 15885865 | 7.23X10^-8^ | A | 0.998 | 0.2 | intergenic |  |
| 29 | 46261156 | 7.28X10^-7^ | G | 0.991 | 0.078 | upstream gene | CHKA |
| 29 | 46261500 | 8.32X10^-9^ | C | 0.98 | 0.064 | upstream gene | CHKA |
| 29 | 46261801 | 8.52X10^-9^ | T | 0.978 | 0.06 | upstream gene | CHKA |
| 29 | 46271567 | 4.48X10^-7^ | C | 0.978 | 0.054 | intergenic |  |
| 29 | 49460479 | 8.62X10^-7^ | C | 0.995 | 0.119 | intergenic |  |
