# Supplementary material for: Variance components for bovine tuberculosis infection and multi-breed genome-wide association analysis using imputed whole genome sequence data
Source: PLoS One. 2019 Feb 14;14(2):e0212067. doi: 10.1371/journal.pone.0212067 (PMC6375599; doi:10.1371/journal.pone.0212067)
Supplement: S6 Table — (DOCX) [file pone.0212067.s007.docx]

**Table S6.** Chromosome (BTA), start position, and end position of each quantitative trait loci region defined in the multi-breed analysis (All) and the within-breed analysis of Charolais bulls (CH), Limousin bulls (LM), or Holstein-Friesian bulls (HO) as well as the quantitative trait loci that overlapped between analyses when quantitative trait loci were triggered by SNPs with P-value < 1 x 10^-5^ that also had a minor-allele frequency of >0.01

| Multi-breed analysis | | | |  | Charolais | | | |  | Limousin | | | |  | Holstein-Friesian | | | |
| --- | --- | --- | --- | --- | --- | --- | --- | --- | --- | --- | --- | --- | --- | --- | --- | --- | --- | --- |
| BTA | Start | End | Overlap |  | BTA | Start | End | Overlap |  | BTA | Start | End | Overlap |  | BTA | Start | End | Overlap |
| 1 | 71,746,607 | 71,761,506 |  |  | 1 | 60,130,721 | 60,502,754 |  |  | 1 | 15,636,606 | 16,357,205 |  |  | 2 | 128,274,603 | 128,288,136 |  |
| 6 | 45,866,075 | 45,943,809 |  |  | 1 | 95,448,118 | 95,448,122 |  |  | 1 | 16,425,458 | 16,716,829 |  |  | 6 | 96,382,367 | 100,623,624 |  |
| 6 | 102,640,308 | 102,691,067 | HO |  | 1 | 122,949,578 | 122,964,495 |  |  | 1 | 52,106,088 | 52,126,573 |  |  | 6 | 100,901,047 | 105,829,695 | All |
| 6 | 116,712,958 | 116,712,958 |  |  | 3 | 55,562,950 | 55,562,950 |  |  | 2 | 106,166,441 | 106,226,068 |  |  | 6 | 116,905,158 | 116,958,237 |  |
| 9 | 72,293,978 | 72,317,521 |  |  | 3 | 119,587,026 | 119,801,645 |  |  | 2 | 134,969,129 | 134,970,054 |  |  | 12 | 19,205,373 | 19,241,878 |  |
| 9 | 89,459,738 | 89,469,653 |  |  | 4 | 11,044,937 | 11,094,902 |  |  | 5 | 36,814,927 | 36,919,834 |  |  | 14 | 67,485,533 | 67,679,648 |  |
| 10 | 92,007,484 | 92,028,789 |  |  | 4 | 26,616,617 | 27,314,890 |  |  | 5 | 46,412,092 | 46,607,669 |  |  | 23 | 19,318,600 | 19,438,098 |  |
| 12 | 70,248,991 | 70,271,532 | LM |  | 4 | 73,770,298 | 74,179,652 |  |  | 5 | 113,892,414 | 113,963,205 |  |  | 23 | 19,442,338 | 19,768,908 | All; CH |
| 15 | 14,595,633 | 14,595,633 |  |  | 5 | 104,560,548 | 104,618,328 |  |  | 6 | 51,564,698 | 51,677,968 |  |  | 23 | 21,910,476 | 23,223,462 | All |
| 16 | 4,041,643 | 4,195,105 |  |  | 7 | 39,547,921 | 39,889,297 |  |  | 6 | 91,065,492 | 91,153,565 |  |  | 23 | 23,650,046 | 23,702,775 |  |
| 16 | 63,052,720 | 63,288,669 |  |  | 7 | 73,255,227 | 73,255,247 |  |  | 7 | 1,760,474 | 1,810,039 |  |  | 23 | 24,414,284 | 24,486,558 |  |
| 17 | 53,109,293 | 53,319,505 |  |  | 7 | 96,658,979 | 96,757,661 |  |  | 8 | 8,846,044 | 8,863,661 |  |  | 23 | 24,665,107 | 25,427,281 | All; CH |
| 17 | 66,216,719 | 66,228,330 |  |  | 8 | 48,501,506 | 48,501,594 |  |  | 8 | 8,934,772 | 8,939,765 |  |  | 23 | 25,496,862 | 25,500,300 |  |
| 20 | 60,858,032 | 60,965,117 |  |  | 8 | 80,331,890 | 81,449,285 |  |  | 8 | 9,030,538 | 9,167,100 |  |  | 23 | 27,303,412 | 27,379,769 |  |
| 22 | 53,919,225 | 54,174,356 |  |  | 9 | 8,673,360 | 8,790,619 |  |  | 8 | 9,297,945 | 9,691,405 |  |  | 23 | 27,828,142 | 27,940,133 |  |
| 22 | 54,222,900 | 54,305,381 |  |  | 9 | 12,464,517 | 12,498,146 |  |  | 8 | 19,060,576 | 19,299,760 |  |  | 23 | 28,567,502 | 28,572,226 |  |
| 23 | 19,441,898 | 19,592,407 | CH; HO |  | 9 | 88,851,309 | 88,962,490 |  |  | 9 | 32,174,386 | 32,365,426 |  |  | 23 | 28,631,608 | 28,671,092 |  |
| 23 | 19,617,330 | 19,662,590 | CH; HO |  | 10 | 65,729,693 | 68,643,175 |  |  | 10 | 26,904,011 | 26,904,192 |  |  | 23 | 32,333,875 | 32,359,891 |  |
| 23 | 22,718,870 | 22,727,379 | HO |  | 10 | 78,522,505 | 79,088,425 |  |  | 11 | 61,736,608 | 61,987,048 |  |  |  |  |  |  |
| 24 | 9,655,417 | 9,659,989 |  |  | 11 | 103,901,444 | 103,963,912 |  |  | 11 | 75,754,617 | 75,865,330 |  |  |  |  |  |  |
| 26 | 8,476,143 | 8,476,151 |  |  | 12 | 14,241,927 | 14,297,967 |  |  | 12 | 3,533,989 | 4,266,356 |  |  |  |  |  |  |
|  |  |  |  |  | 13 | 4,609,784 | 4,659,519 |  |  | 12 | 13,527,985 | 13,594,835 |  |  |  |  |  |  |
|  |  |  |  |  | 13 | 20,212,513 | 20,212,513 |  |  | 12 | 69,119,522 | 72,281,718 | All |  |  |  |  |  |
|  |  |  |  |  | 13 | 51,321,579 | 51,373,971 |  |  | 13 | 41,374,369 | 41,556,095 |  |  |  |  |  |  |
|  |  |  |  |  | 14 | 34,653,670 | 35,206,179 |  |  | 14 | 3,646,328 | 3,725,200 |  |  |  |  |  |  |
|  |  |  |  |  | 15 | 37,486,126 | 37,486,166 |  |  | 14 | 7,498,070 | 7,498,070 |  |  |  |  |  |  |
|  |  |  |  |  | 15 | 73,764,997 | 73,764,997 |  |  | 15 | 45,106,067 | 45,309,857 |  |  |  |  |  |  |
|  |  |  |  |  | 15 | 73,765,014 | 73,765,026 |  |  | 16 | 65,107,501 | 65,122,002 |  |  |  |  |  |  |
|  |  |  |  |  | 16 | 28,734,469 | 28,747,292 |  |  | 16 | 67,356,754 | 67,417,818 |  |  |  |  |  |  |
|  |  |  |  |  | 16 | 79,771,666 | 79,790,250 |  |  | 16 | 67,656,222 | 67,656,222 |  |  |  |  |  |  |
|  |  |  |  |  | 18 | 65,212,743 | 65,220,520 |  |  | 16 | 68,037,032 | 68,053,867 |  |  |  |  |  |  |
|  |  |  |  |  | 19 | 7,097,773 | 7,152,252 |  |  | 17 | 16,589,572 | 16,907,959 |  |  |  |  |  |  |
|  |  |  |  |  | 20 | 35,583,242 | 35,598,261 |  |  | 17 | 16,944,623 | 16,945,392 |  |  |  |  |  |  |
|  |  |  |  |  | 21 | 36,826,994 | 36,834,856 |  |  | 17 | 17,725,521 | 17,949,211 |  |  |  |  |  |  |
|  |  |  |  |  | 22 | 26,819,762 | 26,935,138 |  |  | 17 | 29,746,000 | 29,924,897 |  |  |  |  |  |  |
|  |  |  |  |  | 23 | 13,038,089 | 13,190,649 |  |  | 18 | 17,660,029 | 17,698,440 |  |  |  |  |  |  |
|  |  |  |  |  | 23 | 19,512,721 | 19,948,399 | All; HO |  | 19 | 3,819,932 | 4,921,902 |  |  |  |  |  |  |
|  |  |  |  |  | 23 | 24,986,989 | 25,066,670 | HO |  | 19 | 5,599,166 | 5,758,909 |  |  |  |  |  |  |
|  |  |  |  |  | 23 | 25,445,623 | 25,487,577 | HO |  | 20 | 26,137,614 | 26,156,960 |  |  |  |  |  |  |
|  |  |  |  |  | 23 | 27,523,617 | 27,920,763 | HO |  | 20 | 62,443,074 | 62,567,755 |  |  |  |  |  |  |
|  |  |  |  |  | 24 | 12,834,398 | 12,837,556 |  |  | 20 | 67,171,393 | 67,171,393 |  |  |  |  |  |  |
|  |  |  |  |  | 24 | 19,074,798 | 19,124,838 |  |  | 23 | 20,398,891 | 20,406,420 |  |  |  |  |  |  |
|  |  |  |  |  | 24 | 45,112,447 | 45,339,374 |  |  | 23 | 44,141,665 | 44,141,665 |  |  |  |  |  |  |
|  |  |  |  |  | 27 | 19,284,831 | 19,440,696 |  |  | 23 | 47,591,217 | 47,616,745 |  |  |  |  |  |  |
|  |  |  |  |  | 27 | 39,239,522 | 39,264,645 |  |  | 24 | 6,515,379 | 6,638,325 |  |  |  |  |  |  |
|  |  |  |  |  | 29 | 46,260,803 | 46,272,234 |  |  | 25 | 15,936,181 | 15,936,181 |  |  |  |  |  |  |
|  |  |  |  |  |  |  |  |  |  | 25 | 40,278,445 | 40,385,469 |  |  |  |  |  |  |
|  |  |  |  |  |  |  |  |  |  | 26 | 29,210,891 | 29,249,302 |  |  |  |  |  |  |
|  |  |  |  |  |  |  |  |  |  | 27 | 23,448,863 | 23,595,294 |  |  |  |  |  |  |
